# Identification of Tumor‐Specific Surface Proteins Enables Quantification of Extracellular Vesicle Subtypes for Early Detection of Pancreatic Ductal Adenocarcinoma

**DOI:** 10.1002/advs.202414982

**Published:** 2025-03-25

**Authors:** Chen Zhao, Zhili Wang, Hyoyong Kim, Hui Kong, Junseok Lee, Jacqueline Ziqian Yang, Anmin Wang, Ryan Y. Zhang, Yong Ju, Jina Kim, Bing Feng, Dejun Liu, Yating Zhang, Zhenfang Wang, Yandong Zhang, Shujing Guo, Dekang Gao, James S. Tomlinson, Renjun Pei, Jipeng Wan, Stephen J. Pandol, Myung‐Shin Sim, Sungyong You, Ding Ma, Shaohua Lu, Na Sun, Hsian‐Rong Tseng, Yazhen Zhu

**Affiliations:** ^1^ Department of Pathology and Laboratory Medicine David Geffen School of Medicine, University of California, Los Angeles (UCLA) Los Angeles CA 90095 USA; ^2^ California NanoSystems Institute Crump Institute for Molecular Imaging Department of Molecular and Medical Pharmacology David Geffen School of Medicine University of California, Los Angeles (UCLA) Los Angeles CA 90095 USA; ^3^ Cancer Center Renmin Hospital of Wuhan University Wuhan 430060 China; ^4^ CAS Key Laboratory for Nano‐Bio Interface Suzhou Institute of Nano‐Tech and Nano‐Bionics Chinese Academy of Sciences Suzhou 215123 China; ^5^ Department of Pathology Zhongshan Hospital Fudan University Shanghai 200032 China; ^6^ Department of Urology and Computational Biomedicine Cedars‐Sinai Medical Center Los Angeles CA 90048 USA; ^7^ Department of Biliary‐Pancreatic Surgery Renji Hospital Shanghai Jiaotong University Shanghai 200217 China; ^8^ Department of Anesthesiology Nanfang Hospital Southern Medical University Guangzhou 510515 China; ^9^ Department of Pathology School of Basic Medical Sciences Southern Medical University Guangzhou 510515 China; ^10^ Department of General Surgery Second Affiliated Hospital of Soochow University Suzhou 215004 China; ^11^ Department of Surgery David Geffen School of Medicine, University of California, Los Angeles (UCLA) Los Angeles CA 90095 USA; ^12^ School of Nano‐Tech and Nano‐Bionics University of Science and Technology of China Hefei 230026 China; ^13^ Division of Gastroenterology and Hepatology Department of Medicine Cedars‐Sinai Medical Center Los Angeles CA 90048 USA; ^14^ Basic and Translational Pancreatic Research Cedars‐Sinai Medical Center Los Angeles CA 90048 USA; ^15^ Department of Medicine‐Statistics Core University of California, Los Angeles (UCLA) Los Angeles CA 90095 USA; ^16^ Samuel Oschin Comprehensive Cancer Institute Cedars‐Sinai Medical Center Los Angeles CA 90048 USA; ^17^ Jonsson Comprehensive Cancer Center University of California, Los Angeles (UCLA) Los Angeles CA 90095 USA

**Keywords:** cancer diagnosis, extracellular vesicles, liquid biopsy, pancreatic ductal adenocarcinoma

## Abstract

Pancreatic ductal adenocarcinoma (PDAC) is a leading cause of cancer‐related mortality, largely due to late‐stage diagnosis. Reliable early detection methods are critically needed. PDAC‐derived extracellular vesicles (EVs) carry molecules that reflect their parental tumor cells and are detectable in early disease stages, offering a promising noninvasive diagnostic approach. Here, a streamlined PDAC EV Surface Protein Assay for quantifying PDAC EV subpopulations in 300‐µL plasma through a two‐step workflow is presented: i) click chemistry‐mediated EV enrichment using EV Click Beads and trans‐cyclooctene‐grafted antibodies targeting three PDAC EV‐specific surface proteins (MUC1, EGFR, and TROP2), and ii) quantification of enriched PDAC EVs through reverse transcription‐quantitative polymerase chain reaction. The three PDAC EV‐specific surface proteins are identified using a bioinformatics framework and validated on PDAC cell lines and tissue microarrays. The resultant PDAC EV Score, derived from signals of the three PDAC EV subpopulations, demonstrates robust differentiation of PDAC patients from noncancer controls, with area under the receiver operating characteristic curves of 0.94 in the training (*n* = 124) and 0.93 in the validation (*n* = 136) cohorts. This EV‐based diagnostic approach successfully exploits PDAC EV subpopulations as novel biomarkers for PDAC early detection, translating PDAC surface proteins into an EV‐based liquid biopsy platform.

## Graphical abstract

Pancreatic ductal adenocarcinoma (PDAC) extracellular vesicle (EV) surface protein assay operates via a two‐step workflow – Step 1: Click chemistry‐mediated enrichment of PDAC EVs by EV Click Beads, in the presence of one of the three *trans*‐cyclooctene‐grafted PDAC EV‐specific antibodies; Step 2: Quantification of the enriched PDAC EVs by reverse transcription‐quantitative polymerase chain reaction to detect housekeeping *ACTB* mRNA.

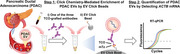

## 1 Introduction

Pancreatic cancer ranks as the third leading cause of estimated cancer‐related mortality in the United States in 2024, with the lowest 5‐year survival rate (13%) among all cancer types and stages.^[^ ^1^ ^]^ Specifically, pancreatic ductal adenocarcinoma (PDAC) accounts for over 90% of pancreatic cancers.^[^ ^2^ ^]^ Due to the nonspecific and vague symptoms of early stage PDAC, over 80% of patients are diagnosed at an advanced stage.^[^ ^3^ ^]^ As a result, only 10%–15% of cases are amenable to surgery, emphasizing the critical need of developing early diagnostic methods for PDAC.

Currently, there is no PDAC screening method available for the general population due to its low prevalence, so diagnosis of PDAC is primarily symptom‐triggered.^[^ ^4^ ^]^ Once symptoms appear, radiographic imaging (CT or MRI) was first employed to visualize the pancreas and its surrounding structures to identify tumors in suspected patients.^[^ ^3^, ^5^ ^]^ Then biopsy such as endoscopic ultrasound (EUS) guided fine‐needle aspiration (FNA) is conducted to confirm the presence of tumor.^[^ ^6^ ^]^ However, these imaging‐based diagnostic methods are not applicable for PDAC early detection. Carbohydrate antigen 19‐9 (CA19‐9) is currently the only widely used biomarker for noninvasive detection of PDAC, but it is not exclusively specific to early stage PDAC and may not be elevated in all patients.^[^ ^4^ ^]^ To meet the need of PDAC early detection, a variety of blood‐based tests, that utilized multiple protein panels,^[^ ^7^ ^]^ cell free‐DNA (cfDNA),^[^ ^7^, ^8^ ^]^ or circular RNA^[^ ^9^ ^]^ were developed. Yet, these approaches often exhibit insufficient sensitivity (ranging from 67% to 78%) to distinguish early stage PDAC from noncancer controls.^[^ ^7^, ^9^ ^]^

Among different liquid biopsy components, extracellular vesicles (EVs) are a heterogeneous group of phospholipid bilayer‐enclosed particles that are released by various cells, particularly tumor cells and those in tumor microenvironment.^[^ ^10^ ^]^ Tumor EVs are present in the bloodstream at relatively early stages of disease^[^ ^11^ ^]^ and their amount escalates throughout disease progression. Tumor EVs protect macromolecules, such as proteins, DNA, RNA, metabolites, and lipids that mirror the tumor of origin. Furthermore, their membranes display surface proteins of their parental tumor, which can be specifically targeted for EV subpopulation enrichment, making them a promising candidate for cancer biomarker development.^[^ ^10^, ^12^ ^]^ Detecting and characterizing tumor EVs, therefore, is considered a promising liquid biopsy technique for noninvasive PDAC detection.^[^ ^8^, ^13^ ^]^ Significant research efforts have focused on immunoaffinity‐based techniques for detecting tumor EVs by targeting specific tumor surface proteins.^[^ ^14^ ^]^ For instance, melanoma cell‐derived exosomes can be enriched using a streptavidin‐labeled beads with monoclonal antibody as the capture agent,^[^ ^15^ ^]^ and ovarian cancer‐derived EVs can be captured and detected on plasmonic gold sensor surfaces.^[^ ^16^ ^]^ However, these methods typically require antibody precoating using various techniques, such as physisorption,^[^ ^17^ ^]^ amide coupling,^[^ ^18^ ^]^ or biotin‐streptavidin interaction,^[^ ^19^ ^]^ in which its efficiency could be impeded by the small number of antigens present on each tumor EV and potential biological interference.^[^ ^20^ ^]^ To overcome these issues, our team has developed innovative technologies that utilize click chemistry for EV enrichment, including EV Click Chips^[^ ^21^ ^]^ and EV Click Beads.^[^ ^22^ ^]^ These technologies offer several improvements: i) the small number of antigens on tumor EVs can be effectively labeled with click motif‐grafted antibodies in a small volume, followed by irreversible enrichment of tumor EV via click chemistry;^[^ ^23^ ^]^ and ii) a highly biorthogonal click reaction, specifically the inverse electron demand Diels–Alder reaction between *trans*‐cyclooctene (TCO) and tetrazine (Tz) motifs, effectively avoids biological interferences commonly encountered with streptavidin‐biotin interactions.^[^ ^24^ ^]^ Our recent studies^[^ ^21^, ^22^, ^25^ ^]^ have further shown that this click chemistry‐based approach not only enhances the specificity and sensitivity of tumor EV enrichment but also provides a more efficient and reproducible method for cancer biomarker development.

**Figure 1:**
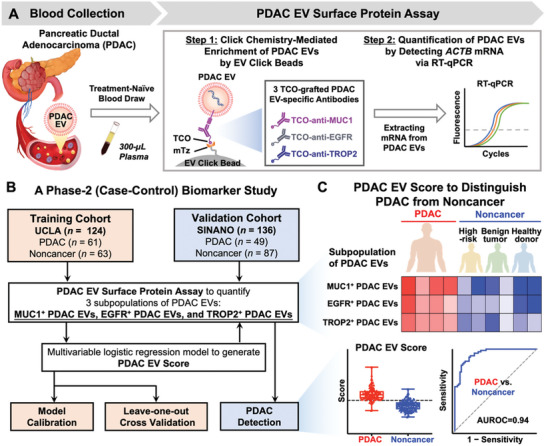
Pancreatic ductal adenocarcinoma (PDAC) Extracellular Vesicle (EV) Surface Protein Assay for noninvasive detection of early stage PDAC. A) PDAC EV Surface Protein Assay is conducted through a two‐step workflow—Step 1: Click chemistry‐mediated enrichment by EV Click Beads in the presence of one of the three trans‐cyclooctene (TCO)‐grafted PDAC EV‐specific antibodies targeting MUC1, EGFR, and TROP2; and Step 2: Quantification of the enriched PDAC EVs by reverse transcription‐quantitative polymerase chain reaction (RT‐qPCR) detecting *ACTB* mRNA. B) A phase 2 (case‐control) biomarker study was developed to assess the diagnostic performance of PDAC EV Surface Protein Assay for distinguishing PDAC from noncancer controls). Plasma samples were collected from a training and a validation cohort. In the training cohort (University of California, Los Angeles [UCLA]), 124 plasma samples were collected from 61 PDAC patients, 63 noncancer controls. In the validation cohort (Suzhou Institute of Nano‐Tech and Nano‐Bionics [SINANO]), 136 plasma samples were collected from 49 PDAC patients, 87 noncancer controls. A logistic regression model was applied to generate PDAC EV Scores from the RT‐qPCR readouts for detecting PDAC. C) The resultant PDAC EV Scores can effectively distinguish PDAC from noncancer. EGFR, epidermal growth factor receptor; EV, extracellular vesicles; mTz, methyltetrazine; MUC1, mucin 1; PDAC, pancreatic ductal adenocarcinoma; RT‐qPCR, reverse transcription‐quantitative polymerase chain reaction; SINANO, Suzhou Institute of Nano‐Tech and Nano‐Bionics; TCO, *trans*‐cyclooctene; TROP2, trophoblast cell‐surface antigen 2; UCLA, University of California, Los Angeles.

**Figure 2:**
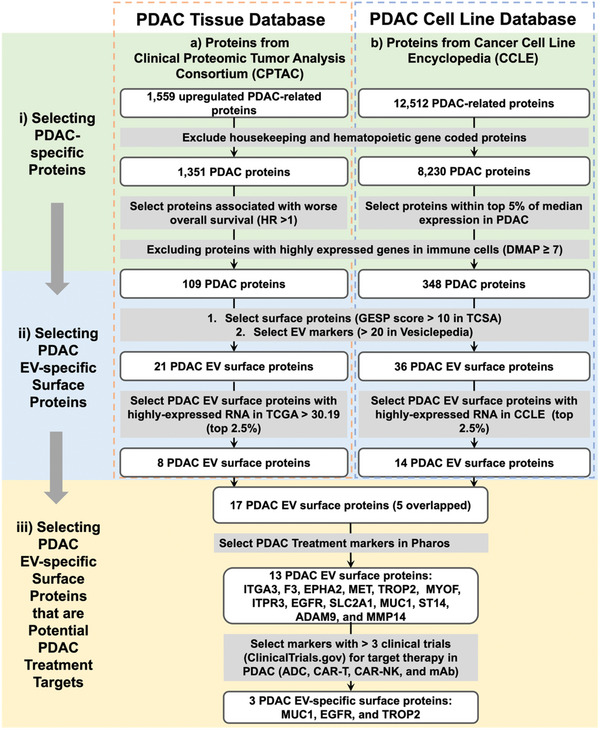
An integrated bioinformatic framework employed for identifying three PDAC EV‐specific surface proteins. The overall PDAC protein markers were compiled from two publicly available datasets: a) Clinical Proteomic Tumor Analysis Consortium (CPTAC) database, which identified 1559 upregulated differential expression proteins in PDAC tissues. b) Cancer Cell Line Encyclopedia (CCLE) protein database, which identified 12512 PDAC‐related proteins in PDAC cell lines. The framework employed sequential filtering steps: i) selection of PDAC‐specific proteins by including highly expressed markers in PDAC and excluding housekeeping and hematopoietic proteins, as well as those markers highly expressed in immune cell expression profiling (DMAP); ii) selection of PDAC EV‐specific surface proteins (TCSA GESP score >10), validating through Vesiclepedia (>10 supporting EV studies) and with high expression in mRNA level (CCLE); and iii) prioritization of three PDAC EV‐specific surface proteins that are potential PDAC treatment targets via Druggable Genome Database (Pharos) and ClinicalTrials.gov. ADC, antibody‐drug conjugate; CAR, chimeric antigen receptor; CCLE, Cancer Cell Line Encyclopedia; DMAP, Differentiation Map; EGFR, epidermal growth factor receptor; EV, extracellular vesicles; CPTAC, Clinical Proteomic Tumor Analysis Consortium; FC, fold change; FDR, false discovery rate; GESP, genes encoding surface protein; HR, hazard ratio; mAb, monoclonal antibody; MUC1, mucin 1; PDAC, pancreatic ductal adenocarcinoma; TCGA, The Cancer Genome Atlas; TCSA, The Cancer Surfacesome Atlas; TROP2, trophoblast cell‐surface antigen 2.

In this study, we developed a streamlined PDAC EV Surface Protein Assay that can enrich and quantify three distinct subpopulations of PDAC EVs (**Figure** **1**A), providing a noninvasive method for detecting early stage PDAC. The assay operates via a two‐step workflow—Step 1: Click chemistry‐mediated enrichment of PDAC EVs by EV Click Beads, in the presence of one of the three TCO‐grafted PDAC EV‐specific antibodies (i.e., TCO‐anti‐MUC1, TCO‐anti‐EGFR, and TCO‐anti‐TROP2); Step 2: Quantification of the enriched PDAC EVs by reverse transcription‐quantitative polymerase chain reaction (RT‐qPCR) to detect housekeeping *ACTB* mRNA. We noted that *ACTB* mRNA is stably expressed across various cell types, developmental stages, cell cycles, and external signals, thus its quantification is commonly used as an alternative for cell counting.^[^ ^26^ ^]^ Therefore, we hypothesized that RT‐qPCR quantification of PDAC EV‐encapsulated *ACTB* mRNA would reflect the number of PDAC EVs, providing a convenient method for the quantification of enriched PDAC EVs. In the phase 1 preclinical studies, the three PDAC EV‐specific surface proteins (i.e., MUC1, EGFR, and TROP2) were thoroughly selected from an integrated bioinformatics framework (**Figure** **2**), followed by the validation through both immunofluorescence (IF) of two PDAC cell lines and immunohistochemistry (IHC) of a 200‐case PDAC tissue microarray (TMA). To explore the use of PDAC EV subpopulations for PDAC early detection, a phase 2 (case‐control) biomarker study was conducted to assess the performance of PDAC EV Surface Protein Assay in a training cohort (*n* = 124) and a validation cohort (*n* = 136), as depicted in Figure 1B. For each study subject, 300‐µL plasma sample was tested by PDAC EV Surface Protein Assay to quantify the three subpopulations of PDAC EVs, including MUC1^+^ PDAC EVs, EGFR^+^ PDAC EVs, and TROP2^+^ PDAC EVs. We then applied a logistic regression model to generate the PDAC EV Scores from reverse transcription‐quantitative polymerase chain reaction (RT‐qPCR) readouts that reflected the quantities of the three PDAC EV subpopulations. The resultant PDAC EV Scores demonstrated excellent diagnostic performance in distinguishing PDAC patients from noncancer controls, with an area under the receiver operating characteristic curve (AUROC) of 0.94 (Figure 1C). Notably, PDAC EV Scores also exhibited remarkable performance in differentiating early stage PDAC from high‐risk patients, with an AUROC of 0.89. These findings underscore the potential of the PDAC EV Surface Protein Assay in the early detection of PDAC and in improving current diagnostic modalities for PDAC surveillance among high‐risk populations.

## 2 Results

### 2.1 Selection of Three PDAC EV‐Specific Surface Proteins Through an Integrated Bioinformatic Framework

To identify PDAC EV‐specific surface proteins for click chemistry‐mediated PDAC EV enrichment, we developed an integrated bioinformatics framework (Figure 2, detailed in the Experimental Section), which began with compiling protein data from the Clinical Proteomic Tumor Analysis Consortium (CPTAC)^[^ ^27^ ^]^ and Cancer Cell Line Encyclopedia (CCLE).^[^ ^28^ ^]^ The selection process of this framework involves three key steps: i) selecting PDAC‐specific proteins with high expression in PDAC or a hazard ratio > 1 for worse overall survival, while excluding housekeeping/hematopoietic proteins^[^ ^26c^ ^]^ and genes highly expressed in immune cells (Differentiation Map ≥ 7);^[^ ^29^ ^]^ ii) selecting PDAC EV‐specific surface proteins with a GESP scores > 10 in the Cancer Surfacesome Atlas (TCSA), involvement in > 20 tumor EV studies (Vesiclepedia),^[^ ^30^ ^]^ and high mRNA expression; and iii) prioritizing PDAC EV‐specific surface proteins that are potential PDAC treatment targets in Pharos^[^ ^31^ ^]^ and are featured in at least three registered clinical trials (ClinicalTrials.gov) for targeted therapy in PDAC (detailed information in Table S1, Supporting Information). This yielded three PDAC EV‐specific surface proteins: MUC1, EGFR, and TROP2, for enriching PDAC EV subpopulations. Among these three PDAC EV‐specific surface proteins, MUC1 and EGFR have been included in EV multimarker panels for PDAC detection in both analytical study^[^ ^32^ ^]^ and clinical validation.^[^ ^33^ ^]^ TROP2 is a ubiquitous and promising target in PDAC,^[^ ^34^ ^]^ emerging TROP2‐targeted agents, focusing on their clinical application and therapeutic efficacy against tumors has been investigated in PDAC patients.^[^ ^35^ ^]^ We are the first to investigate TROP2 as a capture marker in PDAC EVs and validated its performance in early detection of PDAC.

### 2.2 Validation of PDAC EV‐Specific Surface Proteins in PDAC Cell Lines and Tissues

**Figure 3:**
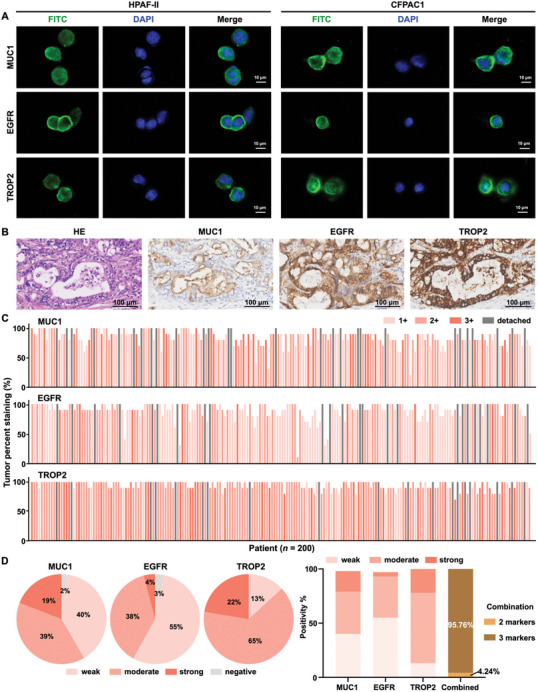
Validation of PDAC EV‐specific surface proteins using PDAC cell lines and PDAC tissue microarray (TMA). A) Representative immunofluorescence (IF) micrographs of the three PDAC EV‐specific surface proteins (MUC1, EGFR, and TROP2) on HPAF‐II and CFPAC1 PDAC cell lines. Blue: DAPI; green: FITC. Scale bar, 10 µm. B) Representative hematoxylin and eosin (H&E) and immunohistochemistry (IHC) images of the three PDAC EV‐specific surface proteins on PDAC TMA slides. Scale bar, 100 µm. C) IHC staining intensity and positive percentages for each marker across PDAC TMA. D) Quantitative IHC scoring is illustrated in pie and bar charts, summarizing the positive percentage of IHC scores for PDAC EV‐specific surface proteins and their combinations. EGFR, epidermal growth factor receptor; FITC, fluorescein isothiocyanate; MUC1, mucin 1; TROP2, trophoblast cell‐surface antigen 2.

Given that EV surface proteins often mirror those of their parental cells, we confirmed MUC1, EGFR, and TROP2 expression on HPAF‐II and CFPAC1 human PDAC cells through IF staining. The representative IF images revealed strong expression of all three surface proteins on both PDAC cell lines (**Figure** **3**A), with predominant localization on the cell membranes. The negative IF staining of the three proteins were observed in white blood cells of HD (Figure S1, Supporting Information).

We further validated the expression of these three surface proteins using IHC in TMA comprising 200 PDAC cases with 96.5% of which are stage I‐II PDAC patients (detailed demographic and clinical information are summarized in Table S2, Supporting Information). The representative hematoxylin and eosin (H&E) and IHC staining images from one case of PDAC tissues are shown in Figure 3B, with moderate membrane expression of MUC1 and strong membrane expression of EGFR and TROP2 on the same area of tumor tissues. IHC staining intensity and positive percentages for each marker across the PDAC TMA was depicted in Figure 3C. The overall quantification of the IHC scoring results of the IHC staining intensity was summarized in Figure 3D. For MUC1, among the 173 evaluable PDAC tissues (excluding 27 cases without tumor tissues or those detached from slides during IHC staining), 19% showed strong staining, 39% with moderate staining, and 40% with weak staining. For EGFR, 4% exhibited strong staining, 38% showed moderate staining, and 55% were weak staining among the 176 evaluable PDAC samples (excluding 24 detached cases). TROP2 had the highest positivity, with 22% strong staining, 65% moderate staining, and 13% weak staining out of the 183 evaluable PDAC samples (excluding 17 detached cases). Notably, all evaluable PDAC samples (165 out of 165) showed positivity for at least two markers, with 95.76% of the samples showing positivity for all three markers. These data highlighted the potential synergy of these three PDAC EV‐specific surface proteins in enriching heterogeneous PDAC EV subpopulations, which forms a solid foundation for their use in the PDAC EV Surface Protein Assay.

### 2.3 Preparation and Characterization of EV Click Beads

**Figure 4:**
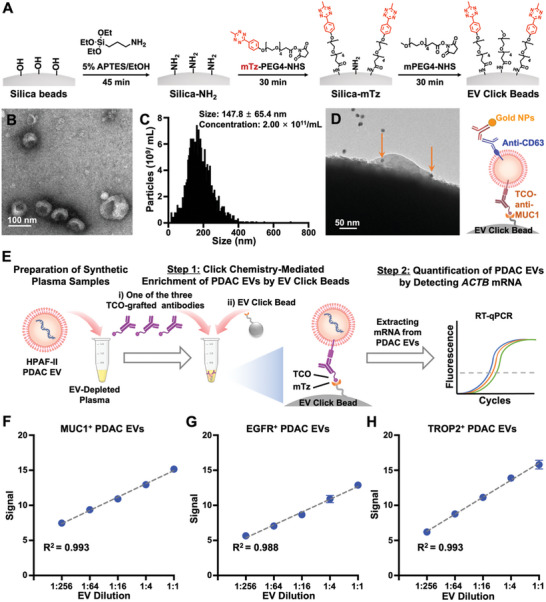
Preparation of EV Click Beads, characterization of PDAC cell‐derived EVs, and linearity study of PDAC EV Surface Protein Assay using synthetic plasma samples. A) Stepwise preparation of EV Click Beads. B) A representative transmission electron microscopy (TEM) image of HPAF‐II EVs. Scale bar, 100 nm. C) Size distribution of HPAF‐II measured by nanoparticle tracking analysis (NTA). D) A representative TEM image of HPAF‐II EVs enriched on an EV Click Bead in the presence of TCO‐anti‐MUC1, followed by immunogold staining with anti‐CD63‐grafted gold nanoparticles (gold arrows). Scale bar, 50 nm. E) A schematic illustration of the workflow developed for linearity study of PDAC EV Surface Protein Assay using synthetic plasma samples. Synthetic plasma samples were prepared by serially spiking HPAF‐II EVs into EV‐depleted HD plasma. Then the two‐step PDAC EV Surface Protein Assay was carried out: Step 1: The synthetic plasma samples were incubated with one of the three PDAC EV‐specific antibodies, followed by enrichment using EV Click Beads; Step 2: The enriched HPAF‐II EVs were quantified by detecting *ACTB* mRNA via RT‐qPCR. F–H) Dynamic linearity ranges of *ACTB* mRNA signals observed for the three subpopulations of PDAC EVs enriched by TCO‐grafted PDAC EV‐specific antibodies, i.e., TCO‐anti‐MUC1 F), TCO‐anti‐EGFR G), or TCO‐anti‐TROP2 H). Signal was defined as 40 − Ct value. APTES, 3‐aminopropyltriethoxysilane; EGFR, epidermal growth factor receptor; EV, extracellular vesicles; mPEG4‐NHS, methoxypolyethylene glycol succinate‐*N*‐hydroxysuccinimide; mTz, methyltetrazine; mTz‐PEG4‐NHS, methyltetrazine‐PEG4‐N‐hydroxysuccinimide; MUC1, mucin 1; NHS, N‐Hydroxysuccinimide; NP, nanoparticle; PDAC, pancreatic ductal adenocarcinoma; RT‐qPCR, reverse transcription‐quantitative polymerase chain reaction; TCO, *trans*‐cyclooctene; TROP2, trophoblast cell‐surface antigen 2.

Building on the outstanding performance of our previous click chemistry‐mediated EV enrichment technology, i.e., EV Click Beads,^[^ ^22^, ^25^ ^]^ we have enhanced the stability and extended the storage life time of the beads from 2 weeks to over 3 months. This was achieved through the substitution of tetrazine (Tz) with methyltetrazine (mTz) as the click motif in the improved platform. The detailed synthetic process is depicted in **Figure** **4**A. It starts with the amine‐modification of silica beads (5 µm diameter) using 3‐aminopropyltriethoxysilane (APTES) with silane chemistry. The amine‐modified silica beads were further modified with methyltetrazine‐PEG4‐*N*‐hydroxysuccinimide (mTz‐PEG4‐NHS) ester via NHS ester chemistry, and any residual amine groups were capped with methoxypolyethylene glycol succinate‐*N*‐hydroxysuccinimide (mPEG4‐NHS) ester, resulting in EV Click Beads endowed with antifouling properties. The characterization of EV Click Beads was described in Figure S2 (Supporting Information), which verified the stability and extended storage life time of the beads.

### 2.4 Characterization of PDAC Cell‐Derived EVs

As a model system, PDAC cell‐derived EVs were harvested from the conditioned media of HPAF‐II PDAC cells using ultracentrifugation (detailed in the Experimental Section). The resultant HPAF‐II EVs were characterized by transmission electron microscopy (TEM) and nanoparticle tracking analysis (NTA) following the guidelines of the International Society for Extracellular Vesicles minimal information for studies of extracellular vesicles (MISEV 2023).^[^ ^36^ ^]^ TEM images (Figure 4B) revealed that HPAF‐II EVs possess a characteristic cupped or spherical‐shaped morphology. NTA data (Figure 4C) indicated that the sizes of these HPAF‐II EVs were 147.8 ± 65.4 nm.

### 2.5 Click Chemistry‐Mediated PDAC EV Enrichment onto EV Click Beads

To confirm the click chemistry‐mediated immobilization of HPAF‐II EVs on EV Click Beads, immunogold staining was employed to label the HPAF‐II EVs, which were later visualized by TEM imaging. In brief, HPAF‐II EVs were incubated with TCO‐anti‐MUC1 and then enriched by EV Click Beads. Subsequently, these samples were incubated with nanogolds‐conjugated anti‐CD63 (a representative EV surface protein) and prepared for TEM. The TEM image (Figure 4D) shows multiple 10 nm gold nanoparticles on the HPAF‐II EVs immobilized on EV Click Beads. Collectively, these findings validated that EV Click Beads are capable of click chemistry‐mediated immobilization of PDAC EVs.

### 2.6 Linearity Study for the *ACTB* mRNA in PDAC Cell‐Derived EVs

To verify the hypothesis that quantification of PDAC EV‐encapsulated *ACTB* mRNA would provide an accurate reflection of the number of PDAC EVs, we conducted a linearity study by RT‐digital PCR quantifying *ACTB* mRNA using serially diluted PDAC cell‐derived EVs. A strong linear relationship was observed between *ACTB* mRNA signals and the quantity of PDAC cell‐derived EVs (*R* ^2^ > 0.99, Figure S3, Supporting Information), confirming the reliability of quantifying PDAC EV‐encapsulated *ACTB* mRNA to reflect the number of PDAC EVs.

### 2.7 Validation of PDAC EV Surface Protein Assay Using Synthetic Plasma Samples

We then validated the PDAC EV Surface Protein Assay using synthetic plasma samples, which were prepared by serially spiking 10 µL of HPAF‐II EVs into 90 µL of EV‐depleted HD plasma over the dilution range of 1:1–1:256 (Figure 4E). Then the PDAC EV Surface Protein Assay was carried out through a two‐step process: i) The 100‐µL synthetic plasma samples were incubated with one of the three PDAC EV‐specific antibodies, i.e., TCO‐anti‐MUC1 (100 ng), TCO‐anti‐EGFR (100 ng), and TCO‐anti‐TROP2 (100 ng), followed by EV enrichment using EV Click Beads; and ii) the enriched HPAF‐II EVs were then subjected to RT‐qPCR for the quantification of PDAC EV subpopulations by detecting the *ACTB* mRNA signals. As shown in Figure 4F–H, strong linear correlations were observed between the spiked HPAF‐II EVs and the detected *ACTB* mRNA signals over the dilution range of 1:1 to 1:256, with *R* ^2^ values ranging from 0.988 to 0.993.

### 2.8 Development of the PDAC EV Score for Distinguishing PDAC Patients from Noncancer Controls in the Training Cohort

**Figure 5:**
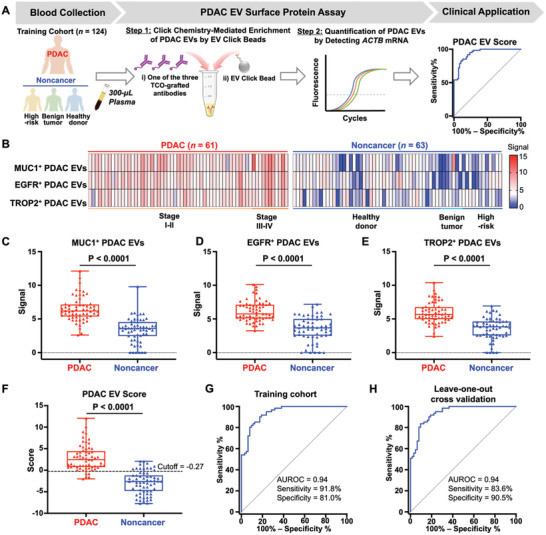
Diagnostic performance of PDAC EV Score for distinguishing PDAC patients from noncancer controls in the training cohort. A) A general workflow illustrating the application of the PDAC EV Surface Protein Assay using plasma samples from 61 PDAC patients and 63 noncancer controls in the training cohort. B) A heatmap summarizing *ACTB* mRNA signals in the three subpopulations of PDAC EVs, including MUC1^+^ PDAC EVs, EGFR^+^ PDAC EVs, and TROP2^+^ PDAC EVs. C–E) Boxplots illustrating significantly higher *ACTB* mRNA signals in the three subpopulations of PDAC EVs among PDAC patients compared to noncancer controls in the training cohort. The signal is represented as 40 – Ct value. F) Boxplots summarizing PDAC EV Scores in PDAC patients and noncancer controls. The dashed line indicates the optimal cutoff of −0.27. G) ROC curve of PDAC EV Score for distinguishing PDAC patients from noncancer controls in the training cohort. H) ROC curve of PDAC EV Score after leave‐one‐out cross‐validation (LOOCV) for distinguishing PDAC patients from noncancer controls in the training cohort. AUROC, area under the receiver operating characteristic curve; EGFR, epidermal growth factor receptor; EV, extracellular vesicles; MUC1, mucin 1; PDAC, pancreatic ductal adenocarcinoma; RT‐qPCR, reverse transcription‐quantitative polymerase chain reaction; TCO, *trans*‐cyclooctene; TROP2, trophoblast cell‐surface antigen 2.

After confirming the linearity of the PDAC EV Surface Protein Assay using synthetic plasma samples, we applied this assay in a training cohort (UCLA) with 61 PDAC patients and 63 noncancer controls (including 8 patients with benign pancreatic tumor, 12 high‐risk patients and 43 HDs). The diagnosis of PDAC and benign pancreatic tumor was confirmed by pathology, and the high‐risk patients were defined according to the American Gastroenterological Association Institute Clinical Practice Update.^[^ ^37^ ^]^ The clinical characteristics and subgroups of the study cohort are detailed in Tables S3 and S4 (Supporting Information). The plasma sample (300 µL) of each study participant was subjected to the two‐step PDAC EV Surface Protein Assay (**Figure** **5**A) to enrich the respective subpopulations of PDAC EVs (i.e., MUC1^+^ PDAC EVs, EGFR^+^ PDAC EVs, and TROP2^+^ PDAC EVs) using EV Click Beads. The quantification of each PDAC EV subpopulation was then obtained by detecting *ACTB* mRNA via RT‐qPCR. The resulting signals of the three subpopulations of PDAC EVs are summarized in Figure 5B. Significantly higher quantities of each PDAC EV subpopulation were observed in the PDAC group compared to the noncancer group, as indicated by the *ACTB* mRNA signals (Figure 5C–E). The respective performance of each PDAC EV subpopulation in distinguishing PDAC patients from noncancer controls was shown in Figure S4 (Supporting Information), with AUROCs ranging from 0.86 to 0.91. Subsequently, *ACTB* mRNA signals in the three PDAC EV subpopulations (i.e., MUC1^+^ PDAC EVs, EGFR^+^ PDAC EVs, and TROP2^+^ PDAC EVs) were incorporated in a multivariable logistic regression model to distinguish PDAC patients from noncancer controls, establishing the PDAC EV Score. The PDAC EV Score was defined as

(1) $$\begin{array}{cc} & \mathrm{PDAC}\;\mathrm{EV}\;\mathrm{Score}=-10.416+0.713\\ & \;\times \mathrm{ACTB}\;\mathrm{mRNA}\;\mathrm{signal}\;\mathrm{of}\;\mathrm{MUC}1^{+}\;\mathrm{PDAC}\;\mathrm{EVs}\\ & \;+0.698\times \mathrm{ACTB}\;\mathrm{mRNA}\;\mathrm{signal}\;\mathrm{of}\;\mathrm{EGF}R^{+}\;\mathrm{PDAC}\;\mathrm{EVs}\\ & \;+0.738\times \mathrm{ACTB}\;\mathrm{mRNA}\mathrm{signal}\mathrm{of}\;\mathrm{TROP}2^{+}\;\mathrm{PDAC}\;\mathrm{EVs} \end{array}$$

The PDAC EV Score demonstrated robust diagnostic performance in distinguishing PDAC patients from noncancer controls in the training cohort (*p* < 0.0001, Figure 5F), with an AUROC of 0.94 (95% CI, 0.91–0.98; Figure 5G). At the optimal cutoff of −0.27, the sensitivity and specificity for PDAC detection were 91.8% and 81.0%, respectively. The positive predictive value (PPV), negative predictive value (NPV), and accuracy were shown in Table S5 (Supporting Information). Additionally, PDAC EV Score was well calibrated with a low Brier score (0.098) and a low mean absolute probability error (0.018) to predict PDAC after 1000 bootstrap resampling (Figure S5, Supporting Information). Leave‐one‐out cross‐validation (LOOCV) of the training cohort confirmed the accuracy of the model with an AUROC of 0.94 (95% CI, 0.90–0.98; Figure 5H), sensitivity of 83.6%, and specificity of 90.5%.

### 2.9 Performance of the PDAC EV Score for Distinguishing PDAC Patients from Noncancer Controls in an Independent Validation Cohort

**Figure 6:**
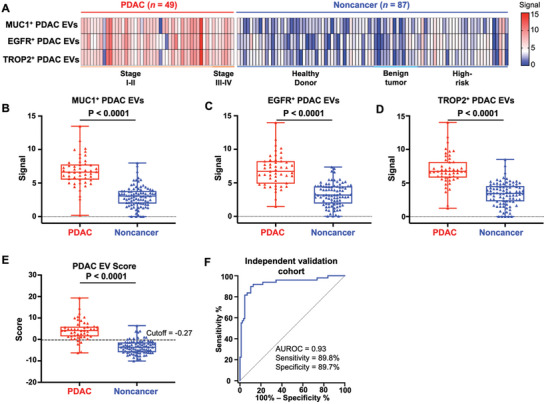
PDAC EV Score for distinguishing PDAC patients from noncancer controls in the validation cohort. A) Heatmaps summarizing *ACTB* mRNA signals in the three subpopulations of PDAC EVs, including MUC1^+^ PDAC EVs, EGFR^+^ PDAC EVs, and TROP2^+^ PDAC EVs. B–D) Boxplots illustrating significantly higher *ACTB* mRNA signals in the three subpopulations of PDAC EVs among PDAC patients compared to noncancer controls in the validation cohort. The signal is represented as 40 – Ct value. E) Boxplots showing PDAC EV Scores observed in PDAC patients compared to noncancer controls in the validation cohort. The dashed line indicates the fixed cutoff of −0.27, which is the same as the training cohort. F) ROC curve of PDAC EV Score for distinguishing PDAC patients from noncancer controls in the validation cohort. AUROC, area under the receiver operating characteristic curve; EGFR, epidermal growth factor receptor; EV, extracellular vesicles; MUC1, mucin 1; PDAC, pancreatic ductal adenocarcinoma; TROP2, trophoblast cell‐surface antigen 2.

After obtaining the PDAC EV Score from the training cohort, we further validated its performance in an independent validation cohort of 49 PDAC patients and 87 noncancer controls recruited at Suzhou Institute of Nano‐Tech and Nano‐Bionics (SINANO), Chinese Academy of Sciences. The clinical characteristics and detailed subgroups information are in Tables S3 and S4 (Supporting Information). The resulting *ACTB* mRNA signals of the three subpopulations of PDAC EVs in the validation cohort are summarized in **Figure** **6**A. The *ACTB* mRNA signals in all three PDAC EV subpopulations (i.e., MUC1^+^ PDAC EVs, EGFR^+^ PDAC EVs, and TROP2^+^ PDAC EVs) were significantly higher in PDAC patients than in noncancer controls (Figure 6B–D, *p* < 0.001). Using the PDAC EV Score formula and the same cut‐off value identified in the training cohort, PDAC EV Scores of the independent validation cohort are calculated and shown in Figure 6E. The PDAC EV Scores maintained excellent performance in distinguishing PDAC patients from noncancer controls, with an AUROC of 0.93 (95% CI, 0.88–0.98; Figure 6F). At the same cutoff value (−0.27) as the training cohort, the sensitivity and specificity were 89.8% and 89.7% for the validation cohort. The PPV, NPV, and accuracy data for validation cohort were shown in Table S6 (Supporting Information). We also explored support vector machines (SVM) as an alternative model to confirm the accuracy of PDAC EV Score (Figure S6, Supporting Information), which indicates similar diagnostic performance with an AUROC of 0.93 (95% CI, 0.87–0.98).

### 2.10 Comparison between PDAC EV Score and Serum CA19‐9 for Detecting PDAC Patients

**Figure 7:**
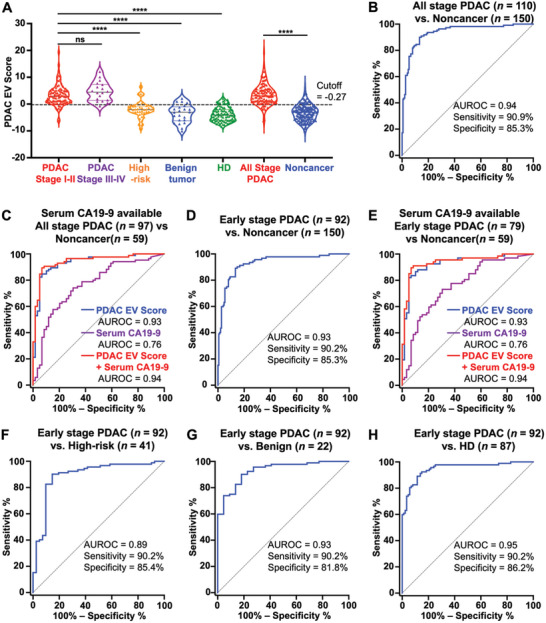
The performance of the PDAC EV Score in all participants and subgroup analyses. A) Violin plots showing the PDAC EV Scores in different subgroups enrolled in this study. B) ROC curve of PDAC EV Score for detecting all stage PDAC (*n = *110) from noncancer controls (*n* = 150). C) ROC curves of PDAC EV Score (blue line), serum CA19‐9 (purple line), and their combination (red line) for detecting serum CA19‐9 available all stage PDAC (*n = *97) from noncancer controls (*n* = 59). D) ROC curve of PDAC EV Score for detecting early stage (stage I–II) PDAC (*n = *92) from noncancer controls (*n* = 150). E) ROC curves of PDAC EV Score (blue line), serum CA19‐9 (purple line) and their combination (red line) for detecting serum CA19‐9 available early stage PDAC (*n = *79) from noncancer controls (*n* = 59). F) ROC curve of PDAC EV Score for detecting early stage PDAC (*n = *92) from high‐risk individuals (*n = *41). G) ROC curve of PDAC EV Score for detecting early stage PDAC (*n = *92) from benign pancreatic tumor (*n = *22). H) ROC curve of PDAC EV Score for detecting early stage PDAC (*n = *92) from HD (*n = *87). ****p < 0.0001, ns: not significant. AUROC, area under the receiver operating characteristic curve; CA19‐9, carbohydrate antigen 19‐9; HD, healthy donor; PDAC, pancreatic ductal adenocarcinoma.

To compare the diagnostic performance of PDAC EV Score and the most commonly used serum biomarker, CA19‐9, we evaluated the diagnostic performance of PDAC EV Score in all participants (training + validation cohorts, *n* = 260) enrolled in this study (**Figure** **7**A). Statistical analysis showed that the PDAC EV Score was not correlated with serum CA19‐9, sex, or age (Figure S7, Supporting Information), which suggested our results would not be biased by these factors. The PDAC EV Score effectively differentiated all stage PDAC patients (*n* = 110) from noncancer controls (*n* = 150) with an AUROC of 0.94 (95% CI, 0.91–0.97; Figure 7B). At the cutoff of −0.27 determined in the training cohort, the sensitivity was 90.9% and the specificity was 85.3%. When compared to serum CA19‐9 (individuals without serum CA19‐9 records were excluded), PDAC EV Score outperformed serum CA19‐9 in distinguishing all‐stage PDAC from noncancer control (AUROC, 0.93; 95% CI, 0.89–0.98 vs AUROC, 0.76; 95% CI, 0.68–0.84; *p* < 0.001) in serum CA19‐9 available participants, with the sensitivity of 92.9% and the specificity of 80.0%. For serum CA19‐9, when applying the standard cutoff (< 36 U mL^−1^),^[^ ^38^ ^]^ it yielded a sensitivity of 74.1% and specificity of 69.5% (Figure 7C). If combining the serum CA19‐9 and the PDAC EV Score, the AUROC increased to 0.94 (95% CI, 0.90–0.98).

### 2.11 The Performance of PDAC EV Score for Detecting Early Stage PDAC

We then assessed the performance of PDAC EV Score in detecting early stage PDAC (stage I–II, *n = *92) from noncancer controls. The PDAC EV Score effectively differentiated early‐stage PDAC (*n* = 92) from noncancer controls (*n* = 150) with an AUROC of 0.93 (95% CI, 0.90–0.96; Figure 7D), a sensitivity of 90.2%, and a specificity of 85.3%. PDAC EV Score outperformed serum CA19‐9 (Figure 7E) in distinguishing early stage PDAC from noncancer controls (AUROC, 0.93; 95% CI, 0.88–0.98 vs AUROC, 0.76; 95% CI, 0.67–0.84; *p* < 0.001). If combining the serum CA19‐9 and the PDAC EV Score, the AUROC increased to 0.94 (95% CI, 0.90–0.98).

The performance of PDAC EV Score remained superior in differentiating early stage PDAC patients from subgroups of noncancer controls. When distinguishing early stage PDAC patients to high‐risk controls, the PDAC EV Score achieved an AUROC of 0.89 (95% CI, 0.83–0.96), with sensitivity of 90.2% and specificity of 85.4% (Figure 7F). PDAC EV Score also demonstrated an AUROC of 0.93 (95% CI, 0.88–0.98) with sensitivity of 90.2% and specificity of 81.8% in distinguishing early stage PDAC from benign pancreatic tumor (Figure 7G). Finally, when distinguishing early stage PDAC patients with HD (*n* = 87), the PDAC EV Score had an AUROC of 0.95 (95% CI, 0.92–0.98) with sensitivity of 90.2% and specificity of 86.2% (Figure 7H). The diagnostic performance of the PDAC EV Score in distinguishing all stages and advanced stage PDAC (stage III–IV) of PDAC from subgroups of benign tumors, HD, or high‐risk patients is summarized in Figure S8 (Supporting Information). The AUROC values range from 0.90 to 0.94 in all stages and 0.94–0.98 in advanced stage PDAC. These results indicated that the performance of the PDAC EV Score remained robust across patient subgroups stratified by PDAC stages and different noncancer control groups.

## 3 Discussion and Conclusion

In this study, we developed a novel PDAC EV Surface Protein Assay for the early detection of PDAC, demonstrating its ability to effectively distinguish PDAC patients from noncancer controls with high sensitivity and specificity. Our assay consists of two essential components: 1) click chemistry‐mediated enrichment of subpopulations of PDAC EVs by EV Click Beads and 2) quantification of enriched PDAC EVs by measuring *ACTB* mRNA using RT‐qPCR. By incorporating the resultant *ACTB* mRNA signals in the three PDAC EV subpopulations in a multivariable logistic regression model, we established PDAC EV Score in the training cohort that distinguished PDAC patients from noncancer controls with a superior AUROC of 0.94. The performance of the PDAC EV Score was reproducible in the validation cohort, with an AUROC of 0.93. The PDAC EV Score also showed satisfactory performance in differentiating early stage PDAC from overall and subpopulations of noncancer participants, including high‐risk individuals, benign pancreatic tumor patients, and HDs, with an AUROC ranging from 0.89 to 0.95. This EV‐based diagnostic approach successfully exploits PDAC EV subpopulations as novel biomarkers for PDAC early detection, effectively translating PDAC surface proteins into an EV‐based liquid biopsy platform.

The majority of existing EV‐based PDAC diagnostics, as detailed in Table S7 (Supporting Information), rely on the use of total EVs. For the upstream enrichment of total EVs, three commonly employed commercially available technologies include ultracentrifugation,^[^ ^32^, ^33^, ^39^ ^]^ water‐exclusion precipitation,^[^ ^40^ ^]^ and affinity chromatography methods.^[^ ^13^, ^41^ ^]^ To address the inherent challenge posed by the heterogeneity of total EVs, the field has begun exploring the use of PDAC EVs for cancer detection. Progress in this area has been relatively limited.^[^ ^8^, ^42^ ^]^ In response to this challenge, our team has developed a novel tumor EV enrichment method leveraging click chemistry, termed EV Click Beads. This innovative approach provides a rapid and irreversible enrichment of EVs. We adopted a click chemistry‐mediated EV enrichment approach by employing an inverse‐electron‐demand Diels‐Alder cycloaddition between mTz and TCO motifs, which increased the interactions between tumor EV antigens and capture antibodies.^[^ ^24b^ ^]^ With a rate constant of 10^2^–10^3^ m ^−1^ s,^−1[^ ^43^ ^]^ the ligation between mTz‐grafted EV Click Beads and TCO‐grafted antibodies is rapid, specific, and irreversible, resulting in the effective immobilization of tumor EVs onto EV Click Beads with enhanced capture sensitivity and specificity.

The study design for developing the PDAC EV Surface Protein Assay was rigorously guided by the established biomarker development principles.^[^ ^44^ ^]^ In the phase 1 preclinical studies, we systematically selected the three PDAC EV‐specific surface proteins (i.e., MUC1, EGFR, and TROP2) through an integrated bioinformatics framework (Figure 2). This bioinformatics framework is distinctive in its ability to select tumor EV‐specific surface proteins by identifying both PDAC‐specific and EV‐specific marker candidates that serve as potential PDAC treatment targets, while effectively eliminating background signals from immune cells. These candidates were further validated through IF analysis of two PDAC cell lines and IHC using a 200‐case PDAC TMA (Figure 3). Additionally, the assay's linearity was evaluated using synthetic plasma samples (Figure 4). Subsequently, we conducted a phase 2 (case‐control) biomarker study to evaluate the performance of the PDAC EV Surface Protein Assay. This study involved a training cohort (*n* = 124) and an independent validation cohort (*n* = 136), ensuring robust assessment of the assay's diagnostic capability. As shown in Table S7 (Supporting Information), only a small portion of the existing EV‐based PDAC diagnostics adopt the established biomarker development principles.

Conventional approaches developed for quantification of enriched EVs include detection of tumor protein concentrations with enzyme‐linked immunosorbent assay (ELISA) or Western Blot,^[^ ^16^, ^45^ ^]^ or enumeration of tumor EV quantities using flow cytometry.^[^ ^15^, ^46^ ^]^ However, these methods often lack the sensitivity to detect EVs at low concentrations in plasma samples or might have a limited accuracy due to the small size of EVs that often fall below the detection threshold.^[^ ^47^ ^]^ In this study, we pioneered a rapid RT‐qPCR‐based methodology to quantify PDAC EVs through detection of EV‐encapsulated *ACTB* mRNA. The membrane‐protected nature of EV‐encapsulated mRNA allows for its stability in PDAC EVs,^[^ ^48^ ^]^ while nonencapsulated mRNA rapidly degrades.^[^ ^49^ ^]^ As a result, quantifying EV‐encapsulated mRNAs could reliably reflect the EV quantity. The selection of *ACTB* as a quantification marker is particularly advantageous due to its consistent expression across diverse cellular contexts, including varying cell types, developmental stages, cell cycle phases, and environmental conditions, making it a robust surrogate marker for cellular quantification.^[^ ^26c^ ^]^ Taken together, detecting *ACTB* mRNA via RT‐qPCR is a reliable approach for the quantification of PDAC EVs, offering practical advantages including cost‐efficiency (about $7 for materials and reagents cost per patient, Table S8, Supporting Information), rapid turnaround time (< 3 h), and minimal sample handling requirements.^[^ ^50^ ^]^ These benefits make the PDAC EV Surface Protein Assay well‐suited for packaging into a standardized kit format, which could leverage existing PCR infrastructure, initially established for COVID‐19 testing, for cancer early detection applications.

The *ACTB* mRNA signals of the three PDAC EV subpopulations (i.e., MUC1^+^ PDAC EVs, EGFR^+^ PDAC EVs, and TROP2^+^ PDAC EVs) were subjected to a multivariable logistic regression model to generate the PDAC EV Score. The PDAC EV Score exhibited remarkable performance in differentiating early stage PDAC and noncancer controls while the performance remained consistent across various noncancer subgroups (Figure 7; and Figure S8, Supporting Information), highlighting the potential of the assay and paving the way for further validation. Our approach specifically enriches PDAC EVs by targeting PDAC EV‐specific surface proteins and quantifies PDAC EV subpopulations while minimizing confounding signals from immune cells or other peripheral sources. As noncancer subgroups (i.e., high‐risk patients, benign tumors, and healthy donors) lack PDAC EVs, their PDAC EV Scores are significantly lower than PDAC patients. This result is consistent with a previous study,^[^ ^33^ ^]^ which also found that signals of EGFR^+^ EV and MUC1^+^ EV were significantly lower in pancreatitis, benign tumor and age‐matched controls. Interestingly, we did not observe significantly increased PDAC EV Scores in advanced stage PDAC compared to early stage PDAC. This may be attributed to the design of our assay, which is specifically intended to differentiate PDAC from noncancer controls and is not optimized for staging. Additionally, the relatively small proportion of advanced stage patients included in our cohorts may have further contributed to this observation. Currently, this assay is designed for PDAC early detection and is not intended for guiding treatment interventions. Nevertheless, the promising performance of this assay may augment the clinical management for PDAC in the future. For example, a positive PDAC EV Score in suspected patients could facilitate the initiation of radiographic imaging and EUS‐FNA for confirmative diagnosis.^[^ ^3^, ^5^, ^6^ ^]^

While our PDAC EV Surface Protein Assay demonstrates significant promise, we acknowledge several limitations. First, the relatively small sample sizes in both cohorts may limit comprehensive subgroup analyses. Second, although we implemented relevant guidelines to minimize potential biases, the nature of a phase 2 case‐control study inherently carries certain bias. To address these constraints, we have planned subsequent phase 3 biomarker studies to further validate the efficacy of the PDAC EV Surface Protein Assay in early detection of PDAC.

By successfully translating tumor tissue surface proteins into EV‐based liquid biopsies, we have developed and validated the PDAC EV Surface Protein Assay, pioneering a convenient approach for PDAC early detection. The assay demonstrated outstanding performance in discriminating PDAC patients from noncancer controls across both training and validation cohorts. These results suggest that the PDAC EV Surface Protein Assay is a promising noninvasive diagnostic tool for early detection of PDAC, with significant potential to improve clinical outcomes for PDAC patients.

## 4 Experimental Section

### Integrated Bioinformatics Framework for Identifying the Three PDAC EV‐Specific Surface Proteins

Two datasets were employed for the selection of overall PDAC EV‐specific surface proteins: a) CPTAC,^[^ ^27^ ^]^ which identified 1559 upregulated PDAC‐related proteins in PDAC tissues (log_2_FC ≥ the 99th percentile of the empirical null distribution for log_2_ median ratio, and false discovery rate (FDR) < 0.05); b) CCLE,^[^ ^28^ ^]^ which identified 12512 PDAC‐related proteins. After excluding housekeeping and hematopoietic proteins,^[^ ^26c^ ^]^ PDAC‐specific proteins were selected based on a hazard ratio (HR) > 1 and *p* value < 0.05 for overall survival^[^ ^29a^ ^]^ or by protein expression level in the top 5%. Following this initial selection, the PDAC‐specific surface proteins were further refined by analyzing their expression levels across 38 distinct human hematopoietic lineages using Differentiation Map (DMAP) data,^[^ ^29b^ ^]^ where proteins with high median expression (DMAP ≥ 7) were excluded to minimize background noise from immune cells. Subsequently, PDAC EV‐specific surface proteins were identified through TCSA data^[^ ^30a^ ^]^ (GESP score > 10) and Vesiclepedia (number of cancer EV studies > 20).^[^ ^30b,c^ ^]^ Next, the top 2.5% of PDAC proteins by RNA expression levels were selected, resulting in 17 PDAC EV surface proteins. To determine whether they are druggable markers, drug information was obtained from the Pharos database (https://pharos.nih.gov/).^[^ ^31^ ^]^ Target Development Levels “Tclin” and “Tchem,” representing clinically validated and preclinical targets respectively, were used to refine the list, narrowing it down to 13 PDAC EV surface proteins. Finally, PDAC EV surface proteins with more than three registered clinical trials of targeted therapies (ADC, CAR‐T, CAR‐NK, and mAb) in PDAC from ClinicalTrials.gov were selected, leading to the identification of three PDAC EV‐specific surface proteins for this study: MUC1, EGFR, and TROP2.

### PDAC Cell Line Culture and PDAC EVs Collection from Cell Culture Medium

PDAC cell lines CFPAC‐1 (CRL‐1918) and HPAF‐II (CRL‐1997) were obtained from the American Type Culture Collection (ATCC). The CFPAC‐1 and HPAF‐II cell lines were cultured in respective Iscove's Modified Dulbecco's Medium (IMDM, Thermo Fisher Scientific, USA) and Eagle's Minimum Essential Medium (EMEM, Thermo Fisher Scientific, USA), supplemented with 10% fetal bovine serum (FBS) and 100 U mL^−1^ penicillin‐streptomycin (Thermo Fisher Scientific, USA). To collect PDAC cell‐derived EVs, HPAF‐II cells were grown to 80% confluence in 18 Nunc EasYFlask Flasks (175 cm^2^, Thermo Fisher Scientific, USA). The culture medium was then replaced with serum‐free medium (13 mL per flask) to induce cell starvation for 24 h. The conditioned medium was collected and centrifuged at 300 g for 10 min, followed by a second centrifugation at 2800 g for 10 min to remove cell debris. The medium was subsequently ultracentrifuged at 100 000 g for 90 min, and the EV pellet was resuspended in 400 µL PBS.

### IF Staining of PDAC EV Surface Proteins

CFPAC‐1 and HPAF‐II cells were stained using an immunocytochemistry (ICC) protocol. Cells were fixed with 4% paraformaldehyde (PFA) for 30 min, then permeabilized with 0.1% Triton X‐100 for 10 min at room temperature and blocked with 2% donkey serum (Jackson ImmunoResearch) for 30 min. The cells were then incubated overnight at 4 °C with the following primary antibodies in PBS containing 2% donkey serum: antihuman MUC1, antihuman EGFR, and antihuman TROP2. Following three washes with PBS, cells were stained with DAPI (Invitrogen, 1:1000 v/v) and a secondary antigoat FITC‐conjugated IgG antibody (Invitrogen, 1:500 v/v). The stained cells were imaged using a Nikon Eclipse 90i fluorescence microscope equipped with a 40× objective lens.

### IHC Staining of PDAC EV‐Specific Surface Proteins on PDAC TMA

The pathological analysis, which included H&E staining, assessment, and IHC staining and scoring on the TMA from 200 PDAC patients at Zhongshan Hospital, Fudan University, Shanghai, was performed by experienced pathologists. The original PDAC samples were fixed in 10% neutral formalin for 24–48 h, embedded in paraffin, and processed into formalin‐fixed paraffin‐embedded (FFPE) blocks following standard pathological procedures. H&E and IHC staining were conducted according to Clinical Laboratory Improvement Amendments (CLIA)‐compliant protocols. Serial sections (4‐µm thick) from TMA blocks were mounted on poly‐l‐lysine‐coated glass slides. Standard IHC staining on these sections was performed using the Ventana Benchmark ULTRA Slide Stainer, following protocols optimized for each antibody through conventional IHC methods. The IHC analyses for MUC1, EGFR, and TROP2 were performed under optimal conditions optimized individually for each antibody. Two experienced pathologists (S.L. & H.K.) independently evaluated and scored the expression of these three proteins on the PDAC tissues in the TMA. Staining intensity was evaluated using a four‐point scale (none, 0; weak, 1+; moderate, 2+; strong, 3+) on digital pathology slides. The IHC scoring was performed based on the positive area and staining intensity, as described in a previous study.^[^ ^51^ ^]^

### Synthesis of EV Click Beads

The EV Click Beads (10 mg, 6 × 10⁸ beads, 2.5 µm in diameter, and 2.0 g cm^−^^3^ density) were first incubated in 2.0 N nitric acid (HNO₃) for 10 min to regenerate hydroxyl groups. Subsequently, they were silanized in an ethanol solution containing 4% v/v (3‐aminopropyl) triethoxysilane (APTES, 25 µL) for 45 min at room temperature. The amino‐functionalized silica microbeads were washed with ethanol to remove unbound silane and then reacted with mTz‐PEG‐NHS ester (0.94 mg, 3.8 mm) in DMSO/PBS (pH = 8.4, 600 µL) for 60 min at room temperature.

### Characterization of EV Click Beads

Zeta potential measurements were conducted at each stage of surface modification of EV Click Beads to confirm successful modification. The bead concentration was maintained at 0.1 mg mL^−1^ in 10% PBS, with each sample replicated in three runs. The amount of mTz on EV Click Beads was determined through back titration using TCO‐Cy5 after incubation with the beads. A standard curve for TCO‐Cy5 was established within the 0–5 µm range in PBS using fluorescence at *λ*_ex = _620 nm/*λ*_em = _680 nm. 0.5 mg of EV Click Beads was dispersed in 200 µL of 5 µm TCO‐Cy5 solution and allowed to react for 1 h at room temperature. The Cy5‐labeled EV Click Beads were magnetically separated, and the fluorescence of the supernatant was measured to determine the amount of unreacted TCO‐Cy5. The Cy5‐labeled EV Click Beads were visualized using fluorescence microscopy (90i, Nikon) in the far‐red channel.

### Preparation of TCO‐Grafted Antibodies

The TCO‐grafted MUC1, EGFR, and TROP2 antibodies were synthesized by incubating TCO‐PEG4‐NHS ester (4 µm, Click Chemistry Tools) with antibodies (1 mg mL^−1^, 20 µL) in BBS (pH = 8.2) for 30 min at room temperature. The resulting TCO‐antibody conjugates were aliquoted and stored at −20 °C until use.

### Characterization of PDAC EVs

NTA was performed using a ZetaView PMX‐120 (Particle‐Metrix, Germany) to determine the size distribution and concentration of PDAC EVs. Samples were diluted in 0.22 µm filtered PBS at appropriate dilution rates ranging from 100 to 10 000‐fold. Each sample was analyzed in triplicate. For electron microscopy, EV samples were fixed in 4% PFA for 30 min and prepared for SEM and TEM. For immunogold staining, fixed EV‐bound beads were incubated with monoclonal antihuman CD63 mouse IgG antibody (Abcam, 1:50 dilution), followed by antimouse nanogold (12 nm, 1:20 dilution), then prepared for TEM imaging.

### Preparation of Synthetic Plasma Samples

For synthetic plasma samples in linearity study of PDAC EV Surface Protein Assay, 10 µL aliquots of PDAC EV pellets were added to 90 µL of EV‐depleted plasma from male healthy donors, with serial dilutions of the spiked HPAF‐II EVs ranging from 1:1 to 1:256. 100 µL synthetic plasma or clinical plasma samples were mixed with one of the three TCO‐grafted PDAC EV‐specific antibodies for 45 min. These samples were incubated with EV Click Beads, followed by centrifugation and washing, and then the three subpopulations of PDAC EVs (i.e., MUC1^+^ PDAC EVs, EGFR^+^ PDAC EVs, and TROP2^+^ PDAC EVs) were subjected to RT‐qPCR for quantification. Prior to testing clinical samples, synthetic PDAC plasma samples using spiked HPAF‐II EVs into HD plasma were employed to determine the performance of EV Click Beads (see the Supporting Methods and Figure S9, Supporting Information).

### PDAC EV Enrichment from Plasma Samples

PDAC EVs in synthetic and clinical plasma samples were enriched using EV Click Beads based on conditions optimized from previous studies.^[^ ^21b^ ^]^ Briefly, 100 ng of TCO‐grafted antibodies (TCO‐anti‐MUC1, TCO‐anti‐EGFR, TCO‐anti‐TROP2) were mixed with 100 µL of plasma sample for 45 min at room temperature. The plasma samples containing TCO‐labeled EVs were incubated with 50 µg of EV Click Beads for 45 min, followed by centrifugation for 90 s. The PDAC EV‐enriched Click Beads were washed three times with washing buffer (TE buffer with 0.05% Tween‐20, pH 8.0) and subsequently analyzed by RT‐qPCR.

### Quantification of PDAC EVs by Detecting ACTB mRNA via RT‐qPCR

Enriched PDAC EVs captured by EV Click Bead were lysed using XpressAmp Lysis Buffer with 1% Thioglycerol (Promega, USA). The lysate was subjected to one‐step RT‐qPCR using PrimeDirect Probe RT‐qPCR Mix (Takara, Japan) with *ACTB* primers and probes, and analyzed using a CFX Duet Real‐Time PCR System (Bio‐Rad, USA).

### Patient Enrollment

Plasma samples of PDAC, high‐risk, and benign tumor patients in the training cohort were purchased from commercial biobanks Proteogenex (USA) and Ontario Tumor Bank (Canada), collected between July 2013 and March 2023. HDs were recruited at UCLA under an IRB‐approved protocol (No. 19‐000857). Plasma samples from all participants in the validation cohort were collected from Renji Hospital affiliated to Shanghai Jiaotong University School of Medicine, China (IRB‐approved protocol: KY2020‐160), and Nanfang Hospital of Southern Medical University, China (IRB‐approved protocol: MR‐44‐24‐032938). Participants provided written informed consent and were at least 18 years old. Diagnostic confirmation of PDAC and benign tumor included histopathology for patients who underwent needle biopsy or surgery. High‐risk patients were clinically diagnosed, and their definitions were according to the American Gastroenterological Association Institute Clinical Practice Update.^[^ ^37^ ^]^ In the study, high‐risk patients include chronic pancreatitis, intraductal papillary mucinous neoplasms (IPMN) and diabetes.

### Clinical Blood Sample Processing

Blood samples were collected in BD Vacutainer plastic tubes with EDTA (BD, Cat. No. 366 643) and processed within 4 h. Plasma was isolated by centrifugation at 530 g for 10 min, followed by 4600 g for 10 min to remove cells and debris. Plasma samples were aliquoted and stored at −80 °C.

### Statistical Analysis

Descriptive statistics are presented as median (interquartile range, IQR) for continuous variables, and as numbers and percentages for categorical variables. Comparisons of continuous variables and categorical variables between groups were performed using the Mann–Whitney U test and Fisher's exact test or chi‐square test, respectively. In this retrospective phase 2 biomarker (case‐control) study, the sample size was calculated to compare AUROCs between PDAC EV Score and serum CA19‐9 using the paired DeLong test. A sample size of 80 (40 PDAC and 40 control) was expected to have 80% power to detect the difference between the AUROCs for the PDAC EV Score versus serum CA19‐9, assuming AUROC = 0.92 for the assay, AUROC = 0.77 for serum CA19‐9 for detecting early stage PDAC,^[^ ^38^ ^]^ when a correlation between the assays of 0.5 was assumed. The power was obtained for a two‐sided test at 0.05 significance level. The multivariate logistic regression analysis was applied in the training cohort to generate the PDAC EV Score for detecting PDAC from noncancer control. Youden's index was employed to identify the optimal cutoff of the PDAC EV Score. Model calibration was assessed using a calibration plot of actual occurrence probability versus prediction. LOOCV was used to estimate the performance of the PDAC EV Score in the training cohort. Sensitivity, specificity, PPV, NPV, and AUROC for the PDAC EV Score to discriminate PDAC from noncancer control were estimated in both the training and validation cohorts. SVM was also employed as an alternative model to confirm the accuracy of PDAC EV Score (Supporting Methods in the Supporting Information). All statistical analyses were conducted using GraphPad Prism (version 9.2.0; GraphPad Software, Inc.) and R Studio (version 4.2.3).

## Conflict of Interest

Dr. Yazhen Zhu is a co‐founder and shareholder in Eximius Diagnostics Corp., Dr. Hsian‐Rong Tseng has financial interests in CytoLumina Technologies Corp., Pulsar Therapeutics Corp., and Eximius Diagnostics Corp.

## Supporting information



Supporting Information

## Data Availability

The data used in this study were sourced from the Clinical Proteomic Tumor Analysis Consortium (https://proteomics.cancer.gov/programs/cptac) and the Cancer Cell Line Encyclopedia (https://sites.broadinstitute.org/ccle), both of which are publicly accessible databases. Reagents employed in this investigation are presented in the Experimental Section. Further details on the protocols and statistical code used in this study are available from the corresponding authors upon request.
