# Supplementary material for: Identification of Tumor‐Specific Surface Proteins Enables Quantification of Extracellular Vesicle Subtypes for Early Detection of Pancreatic Ductal Adenocarcinoma
Source: Adv Sci (Weinh). 2025 Mar 25;12(21):2414982. doi: 10.1002/advs.202414982 (PMC12140334; doi:10.1002/advs.202414982)
Supplement: Supplementary file 1 — Supporting Information [file ADVS-12-2414982-s001.pdf]

ADVANCED SCIENCE
Open Access

Supporting Information

for *Adv. Sci.*, DOI 10.1002/advs.202414982

Identification of Tumor-Specific Surface Proteins Enables Quantification of Extracellular Vesicle Subtypes for Early Detection of Pancreatic Ductal Adenocarcinoma

Chen Zhao, Zhili Wang, Hyoyong Kim, Hui Kong, Junseok Lee, Jacqueline Ziqian Yang, Anmin Wang, Ryan Y. Zhang, Yong Ju, Jina Kim, Bing Feng, Dejun Liu, Yating Zhang, Zhenfang Wang, Yandong Zhang, Shujing Guo, Dekang Gao, James S. Tomlinson, Renjun Pei, Jipeng Wan, Stephen J. Pandol, Myung-Shin Sim, Sungyong You, Ding Ma, Shaohua Lu*, Na Sun*, Hsian-Rong Tseng* and Yazhen Zhu**

Supporting Information

Supporting Methods

Evaluation of the performance of EV Click Beads

To evaluate the performance of EV Click Beads throughout the optimization process, we quantified the copy numbers of specific mRNA in EV cargo to determine the efficiency and specificity of recovered PDAC EVs. Synthetic PDAC plasma samples were prepared by spiking 10 μL of HPAF-II EV solution—EVs derived from a PDAC cell line harboring the *KRAS-G12D* mutation (harvested by ultracentrifugation and analyzed by nanoparticle tracking analysis)—into 90 μL of plasma from a healthy donor (HD) with wild-type *KRAS* (*KRAS-WT*). Following PDAC EV enrichment using EV Click Beads in conjunction with TCO-labeled antibodies targeting PDAC EV-specific surface markers (*i.e.*, MUC1, EGFR, or TROP2), the copy numbers of *KRAS-G12D* and *KRAS-WT* transcripts on EV Click Beads were quantified by reverse transcription-digital polymerase chain reaction (RT-dPCR) (**Figure S9A**).

As HD plasma does not harbor *KRAS-G12D* transcripts, the capture efficiency of HPAF-II EVs using EV Click Bead was calculated by the following equation:

$$Capture\ efficiency\ = \frac{KRAS - G12D\ transcript_{Recovered\ EV}}{KRAS - G12D\ transcript_{Original\ EV}}$$

Here, $KRAS - G12D\ transcript_{Original\ EV}$ and $KRAS - G12D\ transcript_{Recovered\ EV}$ represent the copy numbers of *KRAS-G12D* transcripts in the original 10 μL of HPAF-II EV solution and the recovered HPAF-II EVs on EV Click Beads, respectively.

Given the heterozygosity of *KRAS* in PDAC patients (harboring both mutant and wild-type *KRAS* alleles), we first determined the intrinsic ratio of *KRAS-WT* to *KRAS-G12D* transcripts in HPAF-II EVs across a range of concentrations. As shown in **Figure S9B**, a linear correlation ($y = 0.35x$, $R^2 = 0.990$) was observed. Using the established *KRAS-WT* to *KRAS-G12D* ratio of $n = 0.35$, we calculated the number of *KRAS-WT* transcripts in recovered EVs as follows:

$$KRAS - WT\ transcript_{Recovered\ EV} = KRAS - G12D\ transcript_{Recovered\ EV} \times 0.35$$

The specificity of the HPAF-II EV enrichment using EV Click Beads was calculated as the ratio of the total *KRAS* transcripts (*KRAS-G12D* + *KRAS-WT*) contributed by recovered HPAF-II EVs to the total KRAS transcripts contributed by both enriched HPAF-II EVs (harboring both

KRAS-G12D and *KRAS-WT*) and non-specifically bound background plasma-derived EVs (harboring only *KRAS-WT*). The specificity equation was as follows:

$$Specificity = \frac{KRAS\ transcript_{Recovered\ EV}}{KRAS\ transcript_{Total}}$$

$$= \frac{KRAS - G12D\ transcript_{Recovered\ EV} + KRAS - WT\ transcript_{Recovered\ EV}}{KRAS - G12D\ transcript_{Total} + \ KRAS - WT\ transcript_{Total}}$$

By using the *KRAS-WT* to *KRAS-G12D* ratio of 0.35 from HPAF-II EVs, the equation simplifies to:

$$Specificity = \frac{KRAS - G12D\ transcript_{Recovered\ EV} \times 1.35}{KRAS - G12D\ transcript_{Total} + \ KRAS - WT\ transcript_{Total}}$$

Since HD plasma does not harbor *KRAS-G12D* transcripts, $KRAS - G12D\ transcript_{total}$ was considered equivalent to $KRAS - G12D\ transcript_{recovered\ EV}$. Thus, the specificity was calculated using the copy numbers of *KRAS-G12D* and *KRAS-WT* transcripts of the EV-captured EV Click Beads as follows:

$$Specificity = \frac{KRAS - G12D\ transcript_{total} \times 1.35}{KRAS - G12D\ transcript_{total} + \ KRAS - WT\ transcript_{total}}$$

Support vector machines (SVM)

We trained a SVM model using the svm function in R package e1071. The hyperparameters of kernel width and penalty were fine-tuned in 5-fold cross validation by the caret package. To estimate the accuracy, we performed ROC analysis based on the data from validation cohort using the roc function in the pROC package.

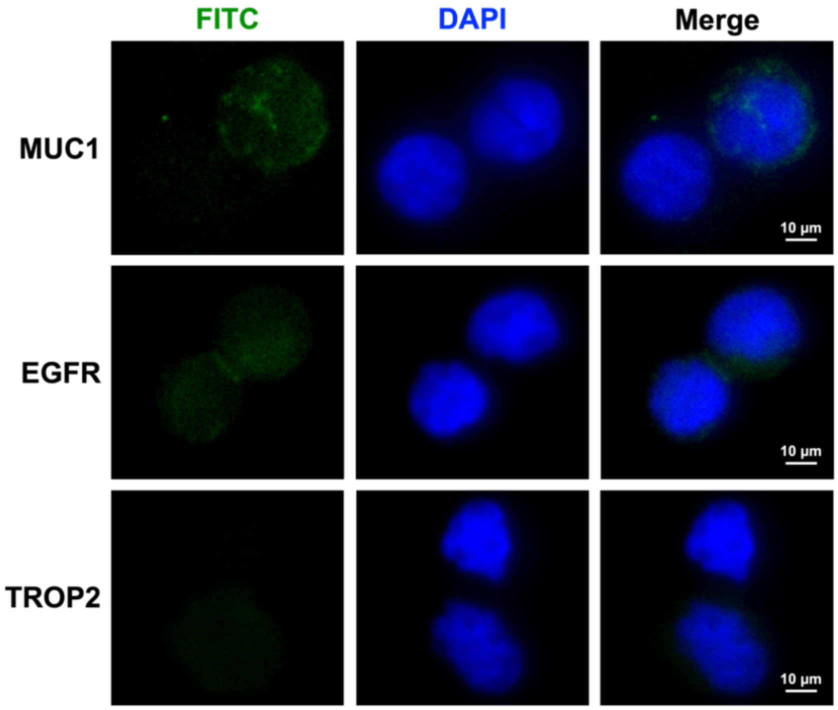

Figure S1. Immunofluorescence (IF) staining images of MUC1, EGFR and TROP2 on white blood cells of healthy donor.

DAPI, 4',6-diamidino-2-phenylindole; EGFR, epidermal growth factor receptor; FITC, fluorescein isothiocyanate; IF, immunofluorescence; MUC1, mucin 1; TROP2, trophoblast cell-surface antigen 2.

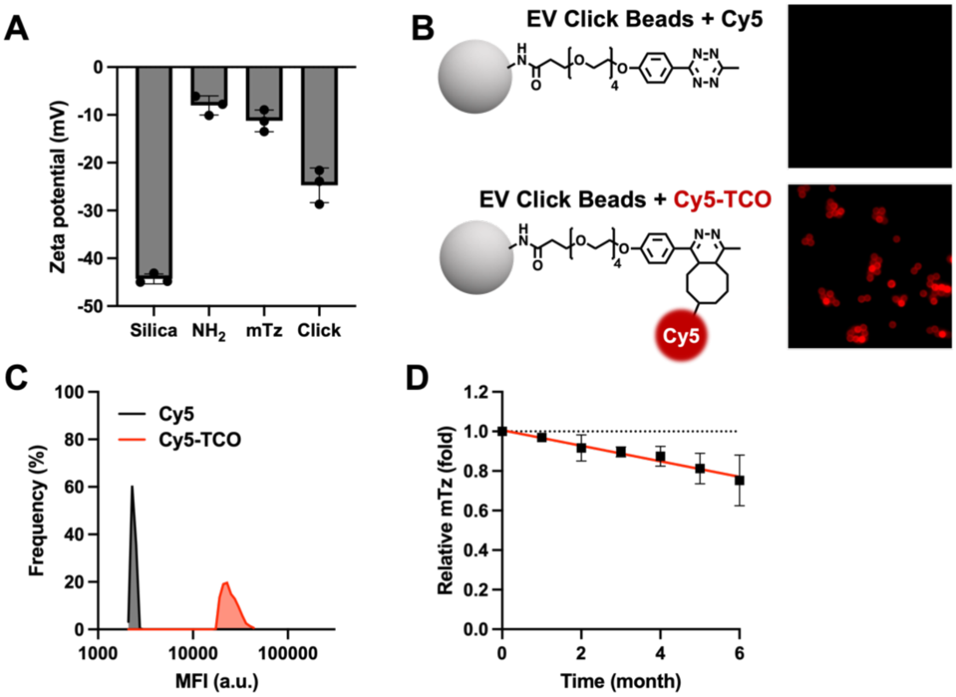

Figure S2. Characterization of EV Click Beads. (A) Zeta potentials of 5 µm silica beads, amine-modified beads (Silica-NH_2), mTz-modified beads (Silica-mTz), and EV Click Beads, indicating the stepwise functional group transformation on the surfaces of the silica beads. **(B)** Fluorescence micrographs of EV Click Beads before and after labeling with TCO-Cy5 or Free Cy5 (scale bar, 20 µm) and **(C)** corresponding histograms of mean fluorescence intensity (MFI). **(D)** Lifetime of the surface mTz group on EV Click Beads measured by Cy5 labeling method.

Cy5, cyanine 5; EV, extracellular vesicles; MFI, mean fluorescence intensity, mTz, methyltetrazine; TCO, *trans*-cyclooctene.

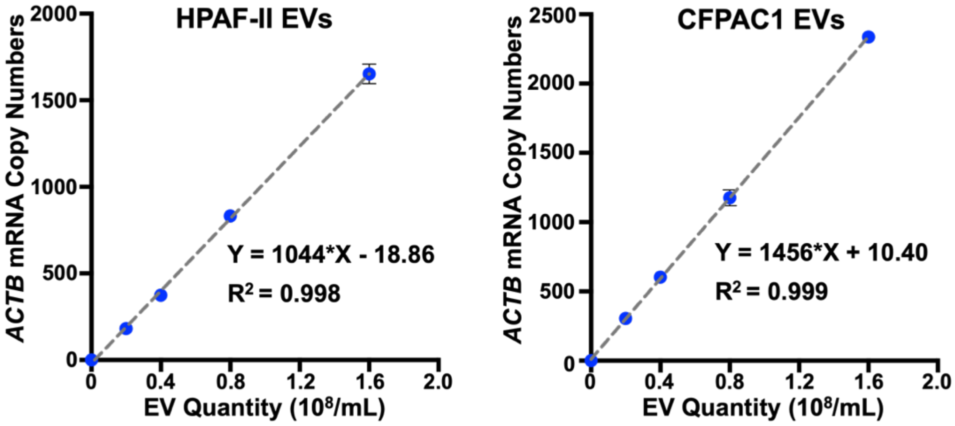

Figure S3. Linearity study for the *ACTB* mRNA in PDAC cell-derived EVs. A strong linear relationship was observed between *ACTB* mRNA copies and the quantity of HPAF-II EVs and CFPAC1 EVs ($R^2 > 0.99$), confirming the reliability of this approach for PDAC EV quantification.

EV, extracellular vesicles; PDAC, pancreatic ductal adenocarcinoma.

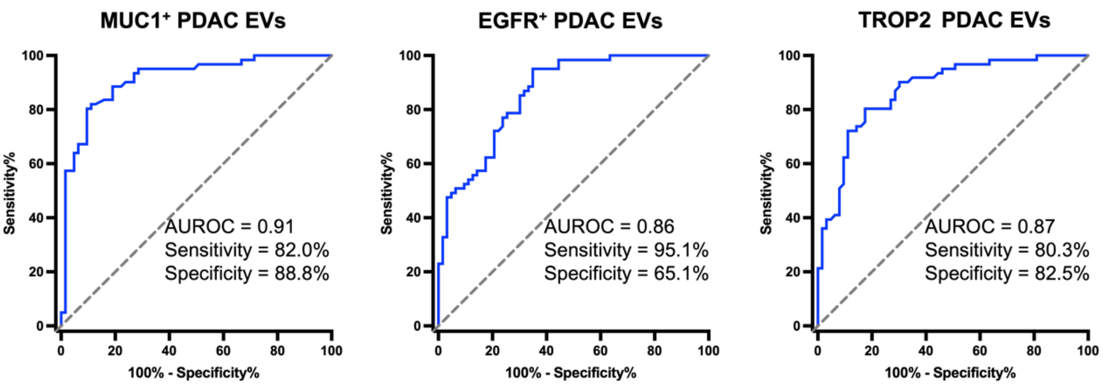

Figure S4. The diagnostic performance of each subpopulation of PDAC EVs.

AUROC, area under receiver operating curve; EGFR, epidermal growth factor receptor; EV, extracellular vesicles; MUC1, mucin 1; TROP2, trophoblast cell-surface antigen 2; PDAC, pancreatic ductal adenocarcinoma.

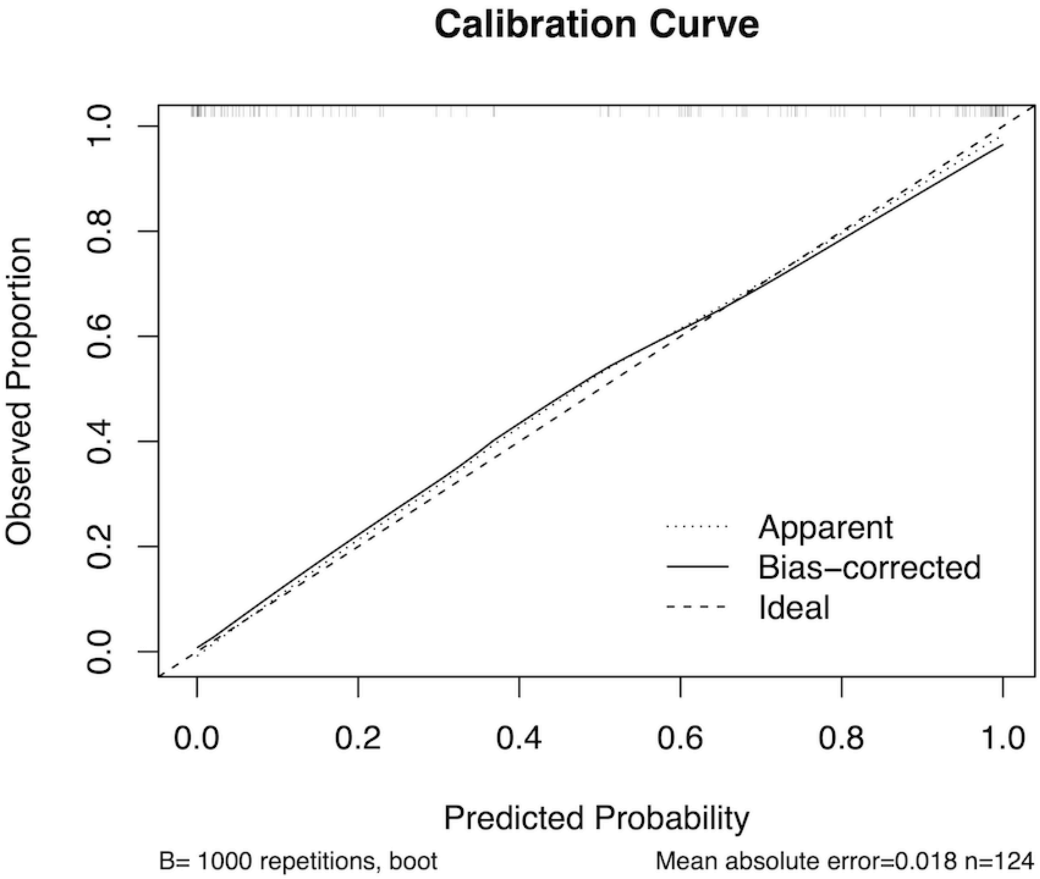

Figure S5. Calibration plot of PDAC EV Score. The apparent calibration curve was performed with the UCLA training cohort of 124 patients with PDAC or noncancer control. The bias-corrected calibration curve was generated by using 1,000 bootstrap resampling of the 124 patients. The predicted probability of PDAC by PDAC EV Score conforms well to the actual probability with a low mean absolute probability error (0.018).

EV, extracellular vesicles; PDAC, pancreatic ductal adenocarcinoma; UCLA, University of California Los Angeles.

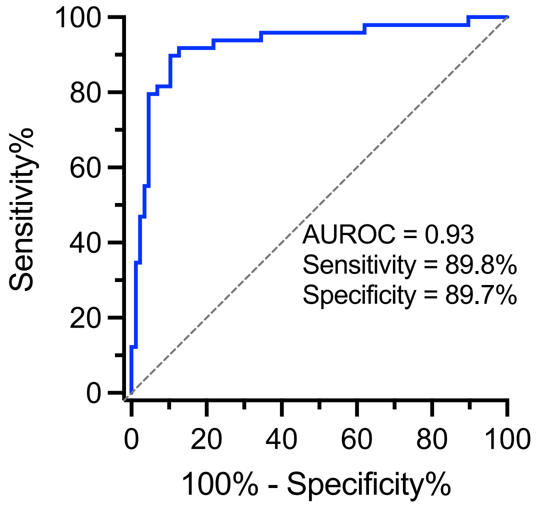

Figure S6. The diagnostic performance of support vector machines (SVM) as an alternative model in distinguishing all stage PDAC from noncancer controls. The SVM model was trained using the training cohort, and result was generated from the validation cohort, achieving a similar diagnostic performance with PDAC EV Score, with an AUROC of 0.93 (95% CI, 0.87 - 0.98).

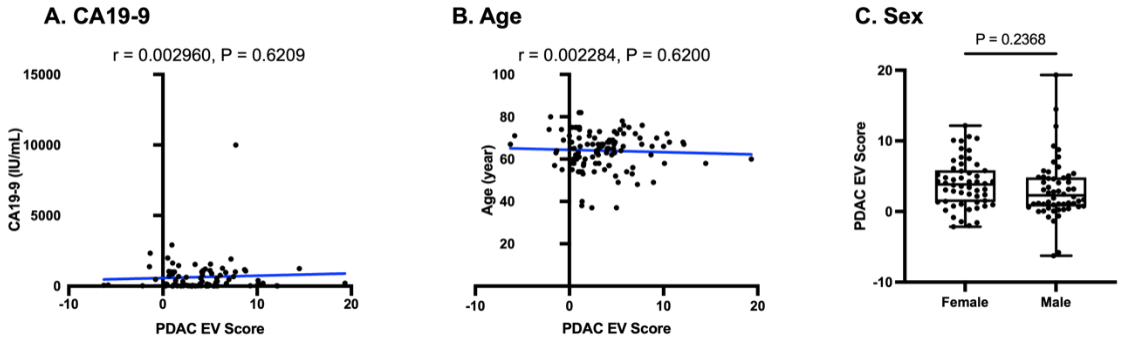

Figure S7. Association between PDAC EV Score and serum CA19-9 level, age and sex. The association between PDAC EV Score and (A) serum CA19-9 level, (B) age and (C) sex of all PDAC patients in the study. Each dot represents a patient.

CA19-9, carbohydrate antigen 19-9; EV, extracellular vesicles; PDAC, pancreatic ductal adenocarcinoma.

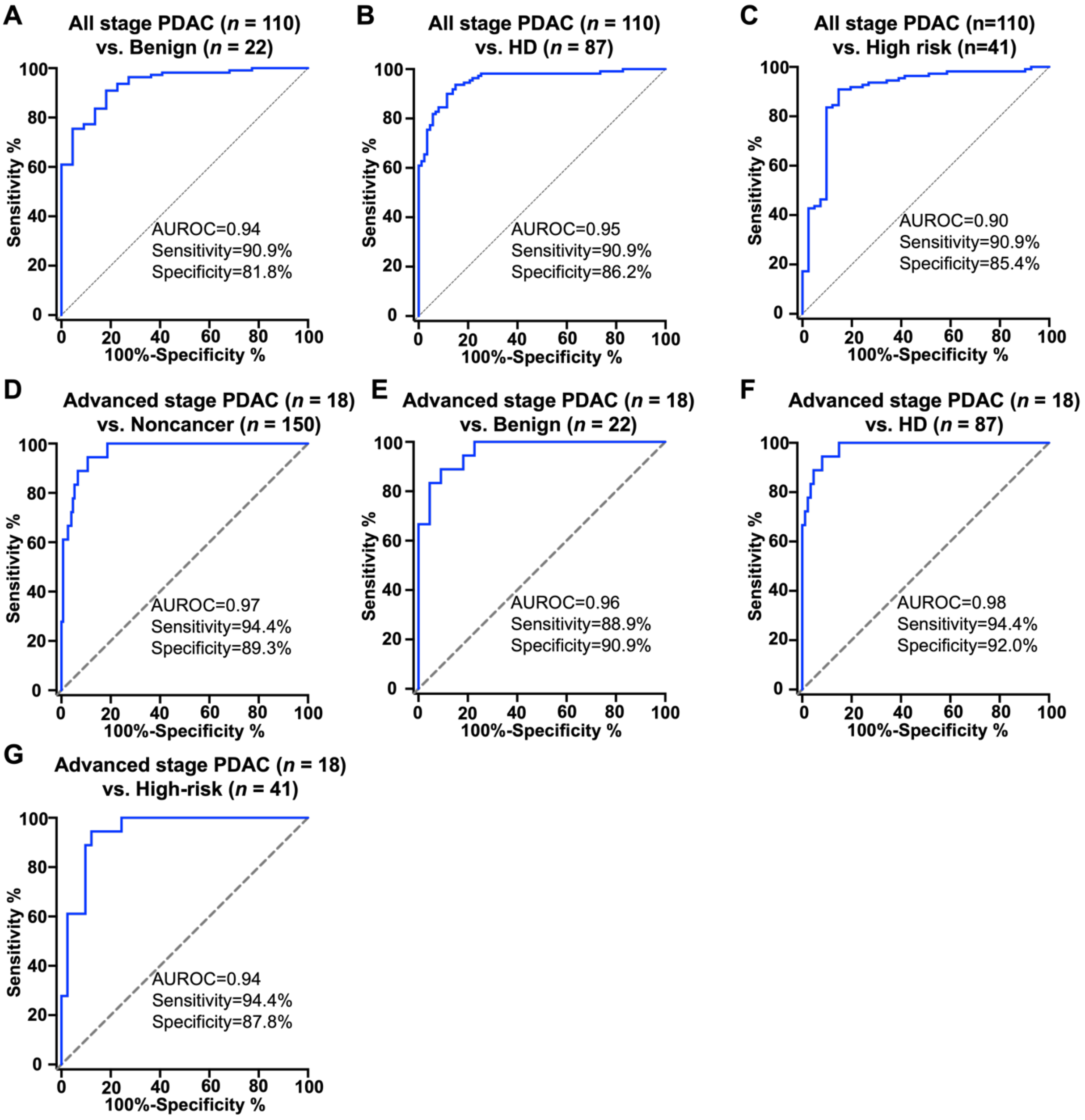

Figure S8. The performance of PDAC EV Score in distinguishing all stage PDAC and advanced stage (Stage III – IV) PDAC. Receiver Operating Characteristic (ROC) curves illustrating the discriminatory ability of PDAC EV Score in distinguishing all stages of PDAC from benign tumors **(A)**, healthy donors (HD) **(B)**, and high-risk patients **(C)**. Additionally, ROC curves demonstrating the ability of PDAC EV Score to differentiate advanced stage (Stage III – IV) PDAC from noncancer controls **(D)**, benign tumors **(E)**, HD **(F)**, and high-risk patients **(G)**.

AUROC, area under the receiver operating characteristic curve; EV, extracellular vesicles; HD, healthy donors; PDAC, pancreatic ductal adenocarcinoma.

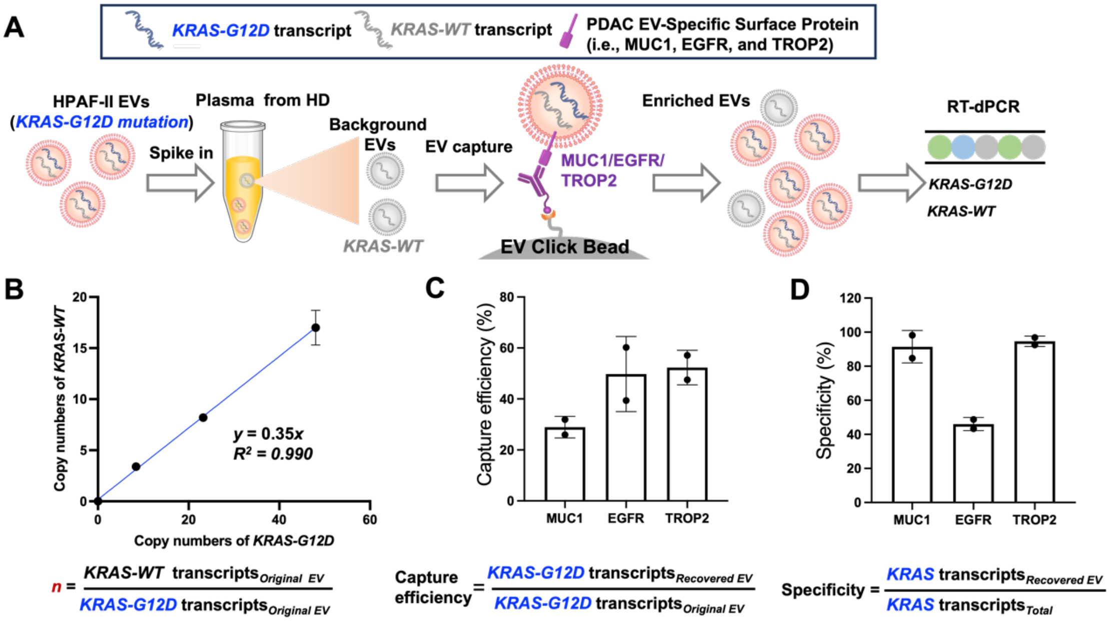

Figure S9. PDAC EV capture efficiency and specificity was calculated by quantifying *KRAS-G12D* and *KRAS-WT* transcripts using synthetic PDAC plasma samples. (A) Synthetic PDAC plasma samples were prepared by spiking 10 µL of HPAF-II EV solution—EVs derived from a PDAC cell line harboring the *KRAS-G12D* mutation (harvested by ultracentrifugation and analyzed by NTA)—into 90 µL of plasma from a healthy donor (HD) with wild-type *KRAS* (*KRAS-WT*). The plasma samples were then incubated with the EV Click Beads with the one of TCO-anti-MUC1, TCO-anti-EGFR, and TCO-anti-TROP2 for PDAC EV enrichment. RT-dPCR was employed to quantify the copy numbers of the *KRAS-G12D* transcripts and *KRAS-WT* transcripts from the enriched EV samples to calculate the efficiency and specificity. *KRAS-G12D* transcripts (a common mutation in the *KRAS* oncogene in PDAC) were solely contributed by HPAF-II EVs, while *KRAS-WT* transcripts were contributed by both enriched HPAF-II EVs and non-specifically captured background HD plasma-derived EVs. **(B)** Verification of the linear correlation between copy numbers of *KRAS-WT* and *KRAS-G12D* transcripts in pure HPAF-II EVs. The ratios between *KRAS-WT* and *KRAS-G12D* transcripts in HPAF-II EVs exhibited a linear correlation ($y = 0.35x$, $R^2 = 0.990$), resulting in the *KRAS-WT* to *KRAS-G12D* ratio of 0.35. Data are presented as mean ± SD ($n = 2$). **(C)** The capture efficiency obtained in the presence of individual TCO-labeled antibody, *i.e.*, TCO-anti-MUC1, TCO-anti-EGFR, or TCO-anti-TROP2. Data are presented as mean ± SD ($n = 2$). **(D)** The specificity of HPAF-II EV enrichment using EV Click Beads was calculated as the ratio of the total *KRAS* transcripts (*KRAS-G12D* + *KRAS-WT*) contributed by enriched HPAF-II EVs to the total KRAS transcripts contributed both recovered HPAF-II EVs (harboring both *KRAS-G12D* and *KRAS-WT*) and non-specifically bound background plasma-derived EVs (harboring only *KRAS-WT*). Data are presented as mean ± SD ($n = 2$).

EV, extracellular vesicle; PDAC, pancreatic ductal adenocarcinoma; HD, healthy donor; RT-dPCR, reverse-transcription digital PCR; TCO, *trans*-cyclooctene.

Table S1. Registered clinical trials (ClinicalTrials.gov) for targeted therapy in PDAC (as of 1/31/2024)

Markers	ADC	CAR-T	CAR-NK	Monoclonal antibody	Registered trials
ITGA3(CD49c)	0	0	0	0	0
F3	0	0	0	0	0
EPHA2	1	0	0	0	1
MET(HGFR)	1	0	0	1	2
TACSTD2(TROP2)	3	0	1	0	4*
MYOF	0	0	0	0	0
ITPR3	0	0	0	0	0
EGFR	0	3	0	8	11
SLC2A1 (GLUT1)	0	0	0	0	0
MUC1	0	4	1	0	5
ST14	0	0	0	0	0
ADAM9	0	0	0	0	0
MPP14	0	0	0	0	0

*not including a TROP2-targeting mRNA-based CAR therapy

Table S2. Demographic and clinical characteristics of PDAC TMA samples.

Characteristic	PDAC (*n* = 200)
**Age, y (IQR)**	67 (41-84)
**Sex**	
Male	108 (54.0%)
Female	92 (46.0%)
**T**	
1	52 (26.0%)
2	113 (56.5%)
3	33 (16.5%)
4	2 (1.0%)
**N**	
0	162 (81.0%)
1	33 (16.5%)
2	5 (12.5%)
**M**	
0	200 (100.0%)
**AJCC Stage**	
I	137 (68.5%)
II	56 (28.0%)
III	7 (3.5%)
**Grade**	
G2	118 (59.0%)
G3	82 (41.0%)

Table S3 Demographic and clinical characteristics of PDAC and noncancer patients in the training cohort and validation cohort.

Characteristic	Training cohort		P value	Validation cohort		P value
	PDAC (n = 61)	noncancer (n = 63)		PDAC (n = 49)	noncancer (n = 87)	
**Age, y (IQR)**	63 (31-82)	52 (21-76)	< 0.0001	67 (52-82)	54 (24-82)	< 0.0001
**Sex**						
Male	34 (55.7%)	37	0.86	20 (40.8%)	52 (59.8%)	0.05
Female	27 (44.3%)	27		29 (59.2%)	35 (40.2%)	
**T**						
1	0 (0.0%)	N/A	N/A	8 (16.3%)	N/A	N/A
2	22 (36.1%)	N/A		26 (53.1%)	N/A	
3	33 (54.1%)	N/A		12 (24.5%)	N/A	
4	6 (9.8%)	N/A		3 (6.1%)	N/A	
**N**						
0	29 (47.6%)	N/A	N/A	33 (67.3%)	N/A	N/A
1	30 (49.2%)	N/A		10 (20.4%)	N/A	
2	1 (1.6%)	N/A		4 (8.2%)	N/A	
Unknown	1 (1.6%)	N/A		2 (4.1%)	N/A	
**M**						
0	54 (88.5%)	N/A	N/A	49 (100.0%)	N/A	N/A
1	7 (11.5%)	N/A		0 (0.0%)	N/A	
**AJCC Stage**						
I	20 (32.8%)	N/A	N/A	24 (49.0%)	N/A	N/A
II	31 (50.8%)	N/A		18 (36.7%)	N/A	
III	4 (6.6%)	N/A		7 (14.3%)	N/A	
IV	6 (9.8%)	N/A		0 (0.0%)	N/A	
**Grade**						
G1	3 (4.9%)	N/A	N/A	1 (2.0%)	N/A	N/A
G2	43 (70.5%)	N/A		30 (61.2%)	N/A	
G3	2 (3.3%)	N/A		15 (30.6%)	N/A	
Unknown	13 (21.3%)	N/A		3 (6.1%)	N/A	
**Serum CA19-9** [U/mL, median (IQR)]	243.00 [1] (7.90 - 2001)	42.35 [2] (1.26 - 1729.85)	0.01	77.45 [3] (0.6 - 10000)	13.00 [4] (2.46 - 2209.41)	0.17

[1] n = 51 available; [2] n = 31 available; [3] n = 46 available; [4] n = 28 available.

Table S4 Demographic and clinical characteristics of noncancer patients in the training cohort and validation cohort

1. Healthy Donor

**Characteristic**	**Training Cohort (*n* = 43)**	**Validation Cohort (*n* = 44)**
**Age,y**	52 (21 - 76)	51.5 (24 - 82)
**Sex**		
Female	30 (69.8%)	26 (59.1%)
Male	13 (30.2%)	18 (40.9%)

2. Benign Tumor

**Characteristic**	**Training Cohort (*n* = 8)**	**Validation Cohort (*n* = 14)**
**Age,y**	57.5 (33 - 68)	51.5 (35 - 70)
**Sex**		
Female	6 (75.0%)	10 (71.4%)
Male	2 (25.0%)	4 (28.6%)
**Pathology**		
papillary mucinous cystadenoma	1 (12.5%)	0 (0.0%)
cyst	2 (25.0%)	1 (7.1%)
lipogranuloma	1 (12.5%)	1 (7.1%)
mucinous cystadenoma	2 (25.0%)	4 (28.6%)
serous cystadenoma	2 (25.0%)	7 (50.0%)
hamartoma	0 (0.0%)	1 (7.1%)

3. High-risk patients

3.1 Chronic pancreatitis

**Characteristic**	**Training Cohort (*n* = 12)**	**Validation Cohort (*n* = 2)**
**Age,y**	52.5 (33 - 61)	55.5 (40 - 71)
**Sex**		
Female	3 (25.0%)	0 (0.0%)
Male	9 (75.0%)	2 (100.0%)

3.2 Intraductal papillary mucinous neoplasms (IPMN)

Characteristic	Validation Cohort (*n* = 7)
**Age,y**	64 (42 - 69)
**Sex**	
Female	5 (71.4%)
Male	2 (28.6%)

3.3 Diabetes

Characteristic	Validation Cohort (*n* = 20)
**Age, y**	64 (34 - 77)
**Sex**	
Female	11 (55.0%)
Male	9 (45.0%)
**HbA1C** [mmol/mol, median (IQR)]	8.75 (7.7 - 9.9)

Table S5. Confusion matrix for PDAC EV Score in the training cohort (*n* = 124).

-	**PDAC EV Score** (cutoff = -0.27)		-
Actual	Predicted PDAC	Predicted Noncancer	-
PDAC (*n* = 61)	56	5	Sensitivity = 91.8%
Noncancer (*n* = 63)	12	51	Specificity = 81.0%
-	PPV = 82.4%	NPV = 91.1%	Accuracy = 86.3%

Table S6. Confusion matrix for PDAC EV Score in the validation cohort (*n* = 136).

-	**PDAC EV Score** (cutoff = -0.27)		-
Actual	Predicted PDAC	Predicted Noncancer	-
PDAC (*n* = 49)	44	5	Sensitivity = 89.8%
Noncancer (*n* = 87)	9	78	Specificity = 89.7%
-	PPV = 83.0%	NPV = 94.0%	Accuracy = 89.7%

Table S7. Summary of Existing EV-based diagnostics for PDAC early detection

Upstream EV Enrichment Technologies	Downstream Detection	Study Design		Study Cohorts		Performance for Distinguishing Early PDAC from Noncancer
		Phases of Biomarker Development	Independent Validation	Sample Distributions	Including High-risk Population	
**This work–PDAC EV Surface Protein Assay**						
Click chemistry-mediated enrichment of PDAC EVs by EV Click Beads	Detecting *ACTB* mRNA by RT-qPCR	Phase 1/2	Yes	Total: 260<br>Early PDAC: 92<br>Advanced PDAC: 18<br>Noncancer: 150<br>High-risk: 41	Yes	AUROC=0.93
**Group 1.1–Enrichment of total EVs by ultracentrifugation**						
Ultracentrifugation; Affinity column [1]	Detecting GPRC5C and EPS8 proteins by immunoblotting	Phase 1/2	Yes	Total:108<br>Early PDAC: 46<br>Advanced PDAC: 8<br>Noncancer: 54<br>High-risk: 22	Yes	N/A
Ultracentrifugation; Syringe filter [2]	Protein profiling by a multiplexed plasmonic assay	Phase 1/2	Yes	Total: 75<br>Early PDAC: N/A<br>Advanced PDAC: N/A<br>Noncancer: 53<br>High-risk: 8	Yes	N/A
Ultracentrifugation and double filtration/ Precipitation (Total Exosome Isolation Kit) [3]	Detecting miRNA by RT-qPCR	Phase 1	No	Total: 42<br>Early PDAC: 20<br>Advanced PDAC: 12<br>Noncancer: 10<br>High-risk: 0	No	N/A
Ultracentrifugation [4]	Detecting $KRAS^{mut}$ and $P53^{mut}$ protein by a single-EV analysis technique	Phase 1	No	Total: 25<br>Early PDAC: 16<br>Advanced PDAC: 4<br>Noncancer: 5<br>High-risk: 0	No	N/A

Ultracentrifugation; Syringe filter [5]	Detecting *KRAS*^{G12D} and *TP53*^{R273H} mutations by digital PCR	Phase 1	No	Total: 171<br>Early PDAC: N/A<br>Advanced PDAC: N/A<br>Noncancer:123<br>High-risk: 16	Yes	N/A
**Group 1.2−Enrichment of total EVs by water-exclusion precipitation**						
Water-exclusion precipitation (Total Exosome Isolation Kit) [6]	Detecting GPC1 mRNA and GPC1 protein by immune lipoplex nanoparticle biochip assay	Phase 1/2	Yes	Total: 359<br>Early PDAC: 139<br>Advanced PDAC: 145<br>Noncancer: 75<br>High-risk: 36	Yes	AUROC=0.921
Water-exclusion precipitation (miRCURY® Exosome Serum/Plasma Kit) [7]	Proteomic profiling by nanoscale LC/MS	Phase 1	No	Total: 33<br>Early PDAC: N/A<br>Advanced PDAC: N/A<br>Noncancer: 17<br>High-risk: 7	Yes	N/A
**Group 1.3−Enrichment of total EVs by affinity column**						
Affinity column (exoRNeasy Serum/ Plasma Kit) [8]	Detecting EV long RNAs by RNA-seq	No Phase 1<br>Phase 2 only	Yes	Total: 501<br>Early PDAC: 136<br>Advanced PDAC: 148<br>Noncancer: 217<br>High-risk: 100	Yes	AUROC=0.949
Affinity column; Magnetic beads (MagCapture™ Exosome Isolation Kit) [9]	Detecting $CD63^{+}$, $CD41^{+}$ and $CD61^{+}$ EVs by sandwich enzyme-linked immunosorbent assay	No Phase 1<br>Phase 2 only	No	Total: 78<br>Early PDAC: 23<br>Advanced PDAC: 16<br>Noncancer: 39<br>High-risk: 7	No	AUROC=0.858 for detecting $CD63^{+}$ EV
**Group 1.4−Enrichment of total EVs by other methods**						

Verita™ platform (ACE-based isolation) [10]	Detecting protein biomarkers by Luminex's bead-based immunoassay	Phase 2	Yes	Total: 763<br>Early PDAC: 135<br>Advanced PDAC: 0<br>Noncancer: 628<br>High-risk: 77	Yes	Training cohort: AUROC=0.971, Validation cohort: AUROC=0.965
**Group 2–Enrichment of PDAC EVs**						
Track etched magnetic nanopore (TENPO) device [11]	Parallel detection of miRNA, mRNA, cfDNA, *KRAS* mutations, and CA19-9.	Phase 1/2	Yes	Total: 204<br>Early PDAC: 45<br>Advanced PDAC: 47<br>Noncancer: 115<br>High-risk: 28	Yes	N/A
Graphene field-effect transistors (GFET) device [12]	Detecting GPC-1^+ EVs by GFET biosensor	Phase 1	No	Total: 26<br>Early PDAC: 2<br>Advanced PDAC: 16<br>Noncancer: 8<br>High-risk: 1	No	N/A

Note: Early PDAC: PDAC of Stage I-II; LC/MC: liquid chromatography–mass spectrometry; N/A: not available.

Ref.

1. Yoshioka, Y., Shimomura, M., Saito, K., Ishii, H., Doki, Y., Eguchi, H., Nakatsura, T., Itoi, T., Kuroda, M., Mori, M., & Ochiya, T. (2022). Circulating cancer-associated extracellular vesicles as early detection and recurrence biomarkers for pancreatic cancer. *Cancer science*, *113*(10), 3498–3509. https://doi.org/10.1111/cas.15500
2. Yang, K. S., Im, H., Hong, S., Pergolini, I., Del Castillo, A. F., Wang, R., Clardy, S., Huang, C. H., Pille, C., Ferrone, S., Yang, R., Castro, C. M., Lee, H., Del Castillo, C. F., & Weissleder, R. (2017). Multiparametric plasma EV profiling facilitates diagnosis of pancreatic malignancy. *Science translational medicine*, *9*(391), eaal3226. https://doi.org/10.1126/scitranslmed.aal3226
3. Xu, X., Bhandari, K., Xu, C., Morris, K., & Ding, W. Q. (2023). miR-18a and miR-106a Signatures in Plasma Small EVs Are Promising Biomarkers for Early Detection of Pancreatic Ductal Adenocarcinoma. *International journal of molecular sciences*, *24*(8), 7215. https://doi.org/10.3390/ijms24087215
4. Ferguson, S., Yang, K. S., Zelga, P., Liss, A. S., Carlson, J. C. T., Del Castillo, C. F., & Weissleder, R. (2022). Single-EV analysis (sEVA) of mutated proteins allows detection of stage 1 pancreatic cancer. *Science advances*, *8*(16), eabm3453. https://doi.org/10.1126/sciadv.abm3453
5. Yang, S., Che, S. P., Kurywchak, P., Tavormina, J. L., Gansmo, L. B., Correa de Sampaio, P., Tachezy, M., Bockhorn, M., Gebauer, F., Haltom, A. R., Melo, S. A., LeBleu, V. S., & Kalluri, R. (2017). Detection of mutant KRAS and TP53 DNA in circulating exosomes from healthy individuals and patients with pancreatic cancer. *Cancer biology & therapy*, *18*(3), 158–165. https://doi.org/10.1080/15384047.2017.1281499

6. Li, H., Chiang, C. L., Kwak, K. J., Wang, X., Doddi, S., Ramanathan, L. V., Cho, S. M., Hou, Y. C., Cheng, T. S., Mo, X., Chang, Y. S., Chang, H. L., Cheng, W., Tsai, W. N., Nguyen, L. T. H., Pan, J., Ma, Y., Rima, X. Y., Zhang, J., Reategui, E., … Lee, L. J. (2024). Extracellular Vesicular Analysis of Glypican 1 mRNA and Protein for Pancreatic Cancer Diagnosis and Prognosis. *Advanced science (Weinheim, Baden-Wurttemberg, Germany)*, *11*(11), e2306373. https://doi.org/10.1002/advs.202306373

7. Marin, A. M., Batista, M., Korte de Azevedo, A. L., Bombardelli Gomig, T. H., Soares Caldeira Brant, R., Chammas, R., Uno, M., Dias Araújo, D., Zanette, D. L., & Nóbrega Aoki, M. (2023). Screening of Exosome-Derived Proteins and Their Potential as Biomarkers in Diagnostic and Prognostic for Pancreatic Cancer. *International journal of molecular sciences*, *24*(16), 12604. https://doi.org/10.3390/ijms241612604

8. Yu, S., Li, Y., Liao, Z., Wang, Z., Wang, Z., Li, Y., Qian, L., Zhao, J., Zong, H., Kang, B., Zou, W. B., Chen, K., He, X., Meng, Z., Chen, Z., Huang, S., & Wang, P. (2020). Plasma extracellular vesicle long RNA profiling identifies a diagnostic signature for the detection of pancreatic ductal adenocarcinoma. *Gut*, *69*(3), 540–550. https://doi.org/10.1136/gutjnl-2019-318860

9. Odaka, H., Hiemori, K., Shimoda, A., Akiyoshi, K., & Tateno, H. (2022). CD63-positive extracellular vesicles are potential diagnostic biomarkers of pancreatic ductal adenocarcinoma. BMC gastroenterology, 22(1), 153. https://doi.org/10.1186/s12876-022-02228-7

10. Hinestrosa, J. P., Sears, R. C., Dhani, H., Lewis, J. M., Schroeder, G., Balcer, H. I., Keith, D., Sheppard, B. C., Kurzrock, R., & Billings, P. R. (2023). Development of a blood-based extracellular vesicle classifier for detection of early-stage pancreatic ductal adenocarcinoma. *Communications medicine*, *3*(1), 146. https://doi.org/10.1038/s43856-023-00351-4

11. Yang, Z., LaRiviere, M. J., Ko, J., Till, J. E., Christensen, T., Yee, S. S., Black, T. A., Tien, K., Lin, A., Shen, H., Bhagwat, N., Herman, D., Adallah, A., O'Hara, M. H., Vollmer, C. M., Katona, B. W., Stanger, B. Z., Issadore, D., & Carpenter, E. L. (2020). A Multianalyte Panel Consisting of Extracellular Vesicle miRNAs and mRNAs, cfDNA, and CA19-9 Shows Utility for Diagnosis and Staging of Pancreatic Ductal Adenocarcinoma. *Clinical cancer research : an official journal of the American Association for Cancer Research*, *26*(13), 3248–3258. https://doi.org/10.1158/1078-0432.CCR-19-3313

12. Yin, T., Xu, L., Gil, B., Merali, N., Sokolikova, M. S., Gaboriau, D. C. A., Liu, D. S. K., Muhammad Mustafa, A. N., Alodan, S., Chen, M., Txoperena, O., Arrastua, M., Gomez, J. M., Ontoso, N., Elicegui, M., Torres, E., Li, D., Mattevi, C., Frampton, A. E., Jiao, L. R., … Klein, N. (2023). Graphene Sensor Arrays for Rapid and Accurate Detection of Pancreatic Cancer Exosomes in Patients' Blood Plasma Samples. *ACS nano*, *17*(15), 14619–14631. https://doi.org/10.1021/acsnano.3c01812

Table S8. Materials and reagents cost estimation of the cost of single PDAC EV Surface Protein Assay test.

The materials and reagents cost of a single PDAC EV Surface Protein Assay is estimated to be about **$7**	
**Materials and reagents**	**Cost (US$)**
Three antibodies	2.20
EV Click Beads	1.50
Dilution, blocking & washing buffer	0.83
EV lysis buffer	0.60
PCR primer/probe	0.15
PCR reagents	1.73
**Total**	**7.01**
